# Host adaptive immunity deficiency in severe pandemic influenza

**DOI:** 10.1186/cc9259

**Published:** 2010-09-14

**Authors:** Jesus F Bermejo-Martin, Ignacio Martin-Loeches, Jordi Rello, Andres Antón, Raquel Almansa, Luoling Xu, Guillermo Lopez-Campos, Tomás Pumarola, Longsi Ran, Paula Ramirez, David Banner, Derek Cheuk Ng, Lorenzo Socias, Ana Loza, David Andaluz, Enrique Maravi, Maria J Gómez-Sánchez, Mónica Gordón, Maria C Gallegos, Victoria Fernandez, Sara Aldunate, Cristobal León, Pedro Merino, Jesús Blanco, Fernando Martin-Sanchez, Lucia Rico, David Varillas, Veronica Iglesias, Maria Ángeles Marcos, Francisco Gandía, Felipe Bobillo, Begoña Nogueira, Silvia Rojo, Salvador Resino, Carmen Castro, Raul Ortiz de Lejarazu, David Kelvin

**Affiliations:** 1 Infection & Immunity Unit, Hospital Clínico Universitario-IECSCYL, Avda. Ramón y Cajal 3, 47005 Valladolid, Spain; 2 Microbiology & immunology Service, Hospital Clínico Universitario-SACYL, Avda Ramón y Cajal 3, 47005 Valladolid, Spain; 3 Critical Care Department, Joan XXIII University Hospital-SEMICYUC. Mallafre Guasch 4, 43007 Tarragona, Spain; 4 Critical Care Department, area General. Hospital Vall d'Hebron. Institut de Recerca, Vall d'Hebron-UAB. CIBERES- SEMICYUC. Paseo Vall d'Hebron, 119-129, 08035, Barcelona, Spain; 5 Microbiology Service, Hospital Clínic, IDIBAPS, University of Barcelona, Carrer de Casanova 143, 08036, Barcelona, Spain; 6 University Health Network, Medical Discovery Tower, 3rd floor Room 913-916,101 College Street, Toronto, ON M5G 1L7, Canada; 7 Medical Bioinformatics Department. Instituto de Salud Carlos III. Ctra. Majadahonda-Pozuelo Km. 2200 Majadahonda, Madrid, Spain; 8 Critical Care Department, Hospital Universitario La Fe- SEMICYUC, Valencia, Avda Campanar 21, 46009, Spain; 9 Critical Care Department, Hospital Son Llatzer- SEMICYUC, Ctra. Manacor, km 4, 07198 Palma de Mallorca, Spain; 10 Critical Care Department, Hospital N Sra de Valme- SEMICYUC, Carretera Madrid-Cadiz (Pol. Ind. La Palmera), KM 548 41014 Sevilla, Spain; 11 Critical Care Department, Hospital Clínico Universitario-SACYL/SEMICYUC. Avda. Ramón y Cajal 3, 47005 Valladolid, Spain; 12 Critical Care Department, Hospital Virgen del Camino- SEMICYUC, C/DE IRUNLARREA 4, 31008 Pamplona, Spain; 13 Microbiology Service, Hospital Son Llatzer, Ctra. Manacor, km 4, 07198 Palma de Mallorca, Spain; 14 Critical Care Department, Hospital Universitario Rio Hortega-SACYL- SEMICYUC& CIBER de Enfermedades Respiratorias (Instituto de Salud Carlos III). C/Dulzaina N° 2 47012 Valladolid, Spain; 15 Laboratory of Molecular Epidemiology of Infectious Diseases, National Centre of Microbiology, Instituto de Salud Carlos III, Ctra. Majadahonda-Pozuelo Km. 2200 Majadahonda, Madrid, Spain; 16 Microbiology Service. Hospital N Sra de Valme, Carretera Madrid-Cadiz (Pol. Ind. La Palmera), KM 548 41014 Sevilla, Spain; 17 International Institute of Infection and Immunity, Shantou University, 22 Xinling Road, Shantou, Guangdong Province, 515031, PR China; 18 University of Sassari, Viale Pasquale Stanislao Mancini, 5 07100 Sassari SS, Italy

## Abstract

**Introduction:**

Pandemic A/H1N1/2009 influenza causes severe lower respiratory complications in rare cases. The association between host immune responses and clinical outcome in severe cases is unknown.

**Methods:**

We utilized gene expression, cytokine profiles and generation of antibody responses following hospitalization in 19 critically ill patients with primary pandemic A/H1N1/2009 influenza pneumonia for identifying host immune responses associated with clinical outcome. Ingenuity pathway analysis 8.5 (IPA) (Ingenuity Systems, Redwood City, CA) was used to select, annotate and visualize genes by function and pathway (gene ontology). IPA analysis identified those canonical pathways differentially expressed (*P *< 0.05) between comparison groups. Hierarchical clustering of those genes differentially expressed between groups by IPA analysis was performed using BRB-Array Tools v.3.8.1.

**Results:**

The majority of patients were characterized by the presence of comorbidities and the absence of immunosuppressive conditions. pH1N1 specific antibody production was observed around day 9 from disease onset and defined an early period of innate immune response and a late period of adaptive immune response to the virus. The most severe patients (*n *= 12) showed persistence of viral secretion. Seven of the most severe patients died. During the late phase, the most severe patient group had impaired expression of a number of genes participating in adaptive immune responses when compared to less severe patients. These genes were involved in antigen presentation, B-cell development, T-helper cell differentiation, CD28, granzyme B signaling, apoptosis and protein ubiquitination. Patients with the poorest outcomes were characterized by proinflammatory hypercytokinemia, along with elevated levels of immunosuppressory cytokines (interleukin (IL)-10 and IL-1ra) in serum.

**Conclusions:**

Our findings suggest an impaired development of adaptive immunity in the most severe cases of pandemic influenza, leading to an unremitting cycle of viral replication and innate cytokine-chemokine release. Interruption of this deleterious cycle may improve disease outcome.

## Introduction

Pandemic 2009 influenza A(H1N1)(p2009A(H1N1)) viral infections continues to be a public health threat [1]. While the overall case fatality rate is low (< 0.5%), approximately 9 to 31% of hospitalized patients require admission to an intensive care unit (ICU), and 14 to 46% of these severe patients have a fatal outcome [2-5]. Understanding the pathogenic events leading to critical pandemic H1N1disease is important for designing better strategies for prevention and treatment of severe outcomes. Previous studies examining host immune responses in other emerging viruses such as severe acute respiratory syndrome (SARS)-associated coronavirus, suggest that severe disease is characterized by a malfunction of the switch from innate to adaptive immunity in response to the virus [6]. Similar to severe infections caused by H5N1 influenza virus [7] dysregulated cytokine secretion have been described in severe cases of p2009A(H1N1) [8,9]. Infection by pandemic 2009 influenza virus causes defective host responses to *S. pneumoniae *as showed in *ex vivo *cultured peripheral blood mononuclear cells from pandemic 2009 influenza (A/H1N1) patients [10]. In ferrets infected with pandemic influenza virus, recovery from infection and improved clinical signs are paralleled by a switch between the innate and the adaptive phase of host immune responses [11].

The potential for the use of gene signatures to better assess the immunopathology and clinical management of severe viral infections has been widely demonstrated in the past [6,12,13]. By using a systems biology-based approach, we analyzed the response to viral infection following hospitalization of 19 p2009A(H1N1) critically ill patients admitted to seven Spanish intensive care units. Our results indicate that pandemic H1N1 patients with severe respiratory disease and poor outcome are characterized by an impaired activation of those genes participating in the development of the antiviral adaptive response.

## Materials and methods

### Study design, participants and sample collection

Nineteen patients attending the participants' ICUs with primary viral pneumonia during the acute phase of influenza virus illness with acute respiratory distress and unequivocal alveolar opacification involving two or more lobes with negative respiratory and blood bacterial cultures at admission were recruited from 1 November to 31 December 2009. Patients older than 65 years and younger than 18 years were excluded from the study to avoid immaturity/aging of the immune system as confusion factor in the analysis. Only those patients with confirmed H1N1 infection by real-time polymerase chain reaction (PCR) were included in the study (*n *= 19). Serial blood samples for plasma, serum and RNA were collected by using serum, ethylenediaminetetraacetic acid (EDTA) and PaxGene (BD) venous blood vacuum collection following the manufacturer's instructions at days 1, 3/5 and 7 after admission to the ICU, according to a unified protocol for all the participant centers. A pharyngeal sample was collected in parallel. Fifteen healthy volunteers of similar age to the patients were recruited between workers of the University of Valladolid, Spain. A standard survey was employed to collect the clinical data, including history and physical examination, oximetric measurement, hematological, biochemical, radiological and microbiological investigation in all the participant centers. Treatment decisions for all patients, including corticosteroid therapy, were not standardized and were decided by the attending physician. Informed consent was obtained directly from each patient or their legal representative and also from the healthy controls before enrollment. Patient and control identification remained anonymous. Approval of the study protocol in both the scientific and the ethical aspects was obtained from the Scientific Committees for Clinical Research of each one of the participant centers.

Samples were stored at -80°C until cytokine, antibody and RNAm profiling. Attending to timing of seroconversion (production of antibodies against p2009A(H1N1)), day 9 from onset of symptoms was considered as the border between the innate and the adaptive immune response in the patients, establishing two moments in the evolution of the disease: an early phase (from onset of symptoms (day 0) to day 8) and a late phase (from day 9 and above). Patients were divided into two groups, depending on their respiratory status. The MV group needed invasive mechanical ventilation following admission; the NMV group was composed of those patients not needing mechanical ventilation at any moment during hospitalization. Cytokines, gene expression and viral load of MV patients were compared with those of NMV patients in both early and late phases separately. The number of samples analyzed in each phase is detailed in the Additional file 1.

### Virological works

Viral diagnosis was performed on RNA from pharyngeal swabs in the Microbiology Services of the participant hospitals by reverse transcription-polymerase chain reaction (RT-PCR)-based methods using reagents provided free of charge by the Centers for Disease Control (CDC, Atlanta, GA, USA) or purchased from Roche (Basel, Switzerland) (H1N1 detection set). These samples were also assessed by multiplex PCR (Luminex) with the xTAG RVP kit from Luminex-Abbott for coinfection with respiratory syncytial virus, influenza B virus, parainfluenza viruses 1-4, human metapneumovirus, enteroviruses, rhinovirus, adenovirus, bocavirus and coronaviruses NL63, HKU1, 229E, OC43, in accordance with the manufacturer's instructions. Viral load was quantified in both pharyngeal swabs and plasma in the Virology Lab of the WHO-associated center at Hospital Clinic in Barcelona, Spain, as detailed in Additional file 1. Oseltamivir resistance was directly detected in the initial positive pharyngeal swab by RT-PCR and sequencing of a 1296-bp fragment of the neuraminidase gene for the presence of the mutation H274Y by using an ABI 3130XL Genetic Analyzer.

### Hemagglutination inhibition assays (HAI)

HAI assays were performed on a 100-μl aliquot of the samples at University Health Network (UHN), Toronto, Ontario, Canada. The sera were treated with receptor-destroying enzyme (RDE) of *V. cholerae *by diluting one part serum with three parts enzyme and were incubated overnight in a 37°C water bath. The enzyme was inactivated by 30-min incubation at 56°C followed by the addition of six parts 0.85% physiological saline for a final dilution of 1/10. HI assays were performed in V-bottom 96-well microtiter plates (Corning Costar Co., Cambridge, MA, USA) with 0.5% turkey erythrocytes as previously described [14] using inactivated pandemic influenza A/California/07/2009(p2009A(H1N1)) antigens.

### Microarrays

Microarrays were performed at University Health Network (UHN), Toronto, Ontario, Canada. More detailed explanation of microarray assays is provided in Additional file 1. Ingenuity Pathway Analysis 8.5 (IPA) (Ingenuity Systems, Redwood City, CA, USA) was used to select, annotate and visualize genes by function and pathway (gene ontology). IPA analysis identified those canonical pathways differentially expressed (*P *< 0.05) between comparison groups. Hierarchical clustering of those genes differentially expressed between groups by IPA analysis was performed using BRB-Array Tools v.3.8.1 stable release developed by Dr. Richard Simon and the BRB-array tools development team. Resulting microarray data sets have been uploaded at the GEO microarray data repository [GEO:GSE21802] [15]. We verified changes in microarray gene expression using quantitative real-time PCR (QRT-PCR) for representative genes from our analysis (Figure S1 in Additional file 2). Primers specific for human GAPDH mRNA were used to normalize samples.

### Immune mediator profiling

Immune mediator levels in serum were measured in patients and controls by using the multiplex Bio-Rad 27-plex assay (Hercules, CA, USA) in the Infection & Immunity Unit (Hospital Clínico Universitario-IECSCYL, Valladolid, Spain). This system allows for quantitative measurement of 27 different chemokines, cytokines, growth factors and immune mediators while consuming a small amount of biological material. A number of additional soluble mediators were measured by using enzyme-linked inmunosorbent assays (ELISAs): interferon α and β (Verikine kits purchased from Pbl Interferon Source, Piscataway, NJ, USA), IL-23, TGF-β1 (Quantikine kits purchased from R&D Systems, Minneapolis, MN, USA), IL28A (Legend Max kit purchased from BioLegend, San Diego, CA, USA). Immune mediator's concentration of each individual sample was normalized against the median of the concentration of the control group (*n *= 15), and the resultant ratios were compared between groups of patients.

### Statistical analysis

The Mann-Whitney *U *test was employed for cytokine comparison purposes, since the Saphiro Wilk test evidenced absence of normal distribution of the data, and the Levene test demostrated absence of homogeneity of variance in both MV and NMV groups. Correlation studies between cytokine levels, gene expression levels, viral load and clinical parameters were done by calculating the Spearman correlation coefficients. All statistical tests were two-sided, and *P *< 0.05 was considered significant.

## Results

### Clinical characteristics of p2009A (H1N1) Patients

**Figure 1:**
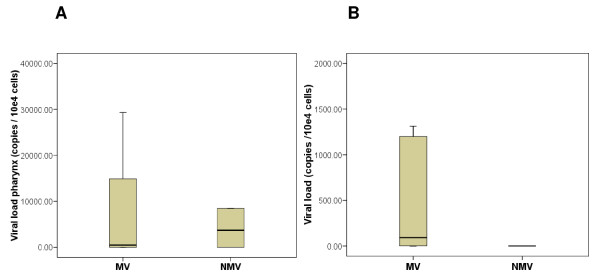
**Viral load in MV and NMV patients in pharynx**. **(a) **Early phase (before day 9 in the course of the disease). **(b) **Late phase (from day 9 in the course of the disease).

All patients were positive for p2009A(H1N1) at admission to the Intensive Care Unit (ICU), with absence of any other respiratory virus in the pharyngeal swabs. None of the patients had received the vaccine against p2009A(H1N1). All patients received oseltamivir therapy by the day of admission to ICU. None of the viral samples examined showed the mutation H274Y conferring resistance to oseltamivir. Twelve patients showed respiratory work severe enough for need of invasive mechanical ventilation at ICU admission (these patients were classified as MV group), while none of the remaining seven were mechanically ventilated during their hospitalization (these patients were classified as NMV group). The most common symptoms at onset were fever > 38°C (cases in MV, cases in NMV) (11, 6), myalgias (8, 6), cough (11, 7) and dyspnea (12, 7). Nine patients of the most severe group and six of the less severe ones had concomitant preexistent conditions (Table 1). None of the patients except one patient of the NMV group who was receiving treatment with bortezomib were under immunosuppressory therapy by the day of admission. Five of 12 MV patients, for four of seven patients in the NMV group, were receiving steroids at the time of sample collection (Table 1). Seven MV patients failed to recover from disease and died with a mean illness duration time of 16.7 and 5.8 days (mean, SD). All NMV patients recovered from p2009A(H1N1) disease. The leading cause of death was primary respiratory failure with refractory hypoxemia in five patients and multiorganic failure in the remaining two patients (Table S1 in Additional file 3). While no differences were found in viral load between MV and NMV patients in the early stage of the disease, MV patients showed significantly higher viral loads than NMV in pharynx in the late stage of the disease (*P *< 0.05) (Figure 1). Three MV patients and one NMV showed detectable viremia at the day of ICU admission, with undetectable virus in plasma afterward. Viral load in pharynx showed a direct correlation with SOFA score (*r *= 0.4) and an inverse one with O_2 _saturation (*r *= -0.3) during the course of the disease. Five MV patients suffered from a bacterial or fungal superinfection at some point during hospitalization (Table 1). Three patients suffering from bacterial superinfection died (Table S1, Additional file 3). Remarkably, none of the patients in the NMV group suffered from bacterial superinfection. All but three patients (two MV and one NMV) had produced antibodies (HAI titers > 1/40) by the last day of sample collection. Seroconversion took place 9.7 (4.6) days from the onset the symptoms in the MV group and 8.8 (1.6) days in the NMV group (mean, SD), with no significant difference between the two groups.

**Table 1:** Clinical and laboratory characteristics of the patients

	MV (*n *= 12)		NMV (*n *= 7)	
Gender (M/F)	7/5		2/5	
Age	45.6 (10.3)		38.5 (13.1)	
Ethnicity	Caucasian (10/12), gipsy (1/12), India (1/12)		Caucasian (5/7), Black (1/7), Magreb (1/7)	
BMI	27.7 (6.3)		27.1 (5.5)	
Pandemic influenza vaccine	0/12		0/7	
Chronic respiratory disease	2/12		1/7	
Chronic renal disease	1/12		2/7	
Cardiovascular disease	1/12		0/7	
Neurological disease	3/12		1/7	
Gastrointestinal disease	2/12		0/7	
Cancer	0/12		1/7	
Obesity (BMI > 30)	3/12		2/7	
Diabetes	1/12		0/7	
Pregnancy	1/12 (32 weeks)		2/7 (31 and 27 weeks)	
Dyslipemia	2/12		0/7	
Alcoholism	1/12		2/7	
Smoker	6/12		3/7	
Fatal outcome/survivors	7/5		0/7	
Duration of symptoms at ICU admission	5.7 (2.3)		6.5 (1.2)	
O_2 _saturation at admission (room air)	88.0 (10.8)		95.3 (2.7)	
Days at ICU	17.5 (22.1)		4.6 (2.3)	
Days at hospital	14.2 (7.4)		11.8 (6.1)	
Days since onset to intubation	6 (2.4)		n.a	
Oseltamivir*	12/12		7/7	
Duration of symptoms before oseltamivir	5.3 (2.3)		6.1 (1.2)	
Days with oseltamivir (days)	8.7 (5.0)		7.1 (2.4)	
Steroids at sampling	5/12		4/7	
Infiltrates in chest X-ray	12/12		7/7	
Progression of infiltrates to all 4 quadrants on chest X-ray	6/12		1/7	
Bacterial/fungal Superinfection [Microbe, sample (resp culture-RC; hemoculture-HC), days from ICU admission to first bacterial isolation, days from symptoms onset to first bacterial isolation]	1. *S. aureus*, *A. fumigatus*, RC, *E. faecalis*, HC, 2,10 2. *S. marcenses*, HC, 7,10 3. *P. aeruginosa*, RC, 5,14 4. *C. albicans*, *S. Marcenses*, RC, 43,52 5. *A. fumigatus*, *C. Albicans*, RC, 6, 12		n.a	

	**MV early**	**MV late**	**NMV early**	**NMV late**

SOFA score	4.0 (3.4)	7.5 (3.1)	3.4 (2.1)	3.5 (2.8)
Creatinine (mg/dl)	0.7 (0.3)	0.7 (0.4)	1.7 (2.8)	1.7 (1.6)
AST (U/liter)	63.5 (34.6)	73.7 (37.3)	73.8 (39.9)	61.8 (78.3)
ALT (U/liter)	26.8 (16.1)	58.8 (38.9)	75.6 (59.9)	76.6 (86.7)
CPK (IU/liter)	221.2 (290.8)	713.0 (640.5)	449.0 (697.8)	152.7 (171.6)
Leucocytes/mm^3^	2594.6 (2907.5)	7923.6 (7100.4)	2917.3 (3723.0)	3524.4 (5208.6)
Neutrophils (%)	52.4 (37.9)	73.3 (27.3)	38.6 (42.9)	34.8 (38.1)
Lymphocytes x10^3 ^/mm^3^	2.2 (2.4)	0.8 (0.6)	0.6 (0.2)	1.1 (0.5)

Data are shown as means with SD where appropriate. N.a., not applicable. R.C., respiratory culture; HC: hemoculture. *Oseltamivir was administered orally or by nasogastric route in accordance with CDC recommendations [31].

### Gene expression profiling

**Figure 2:**
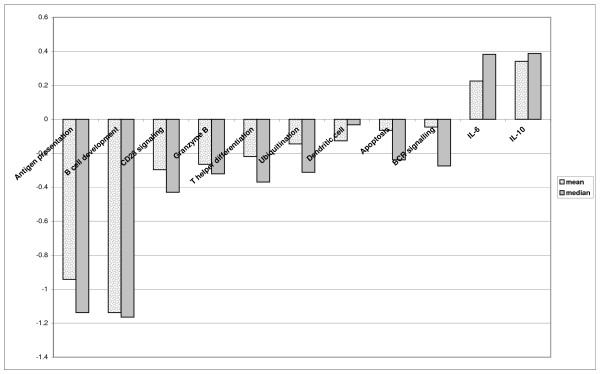
**Histogram depicting the mean and median of the differences in gene expression levels between MV-NMV by intracellular signaling pathways**. (< 0) means that expression in MV < expression in NMV; (> 0) means that expression in MV > expression in NMV).

**Figure 3:**
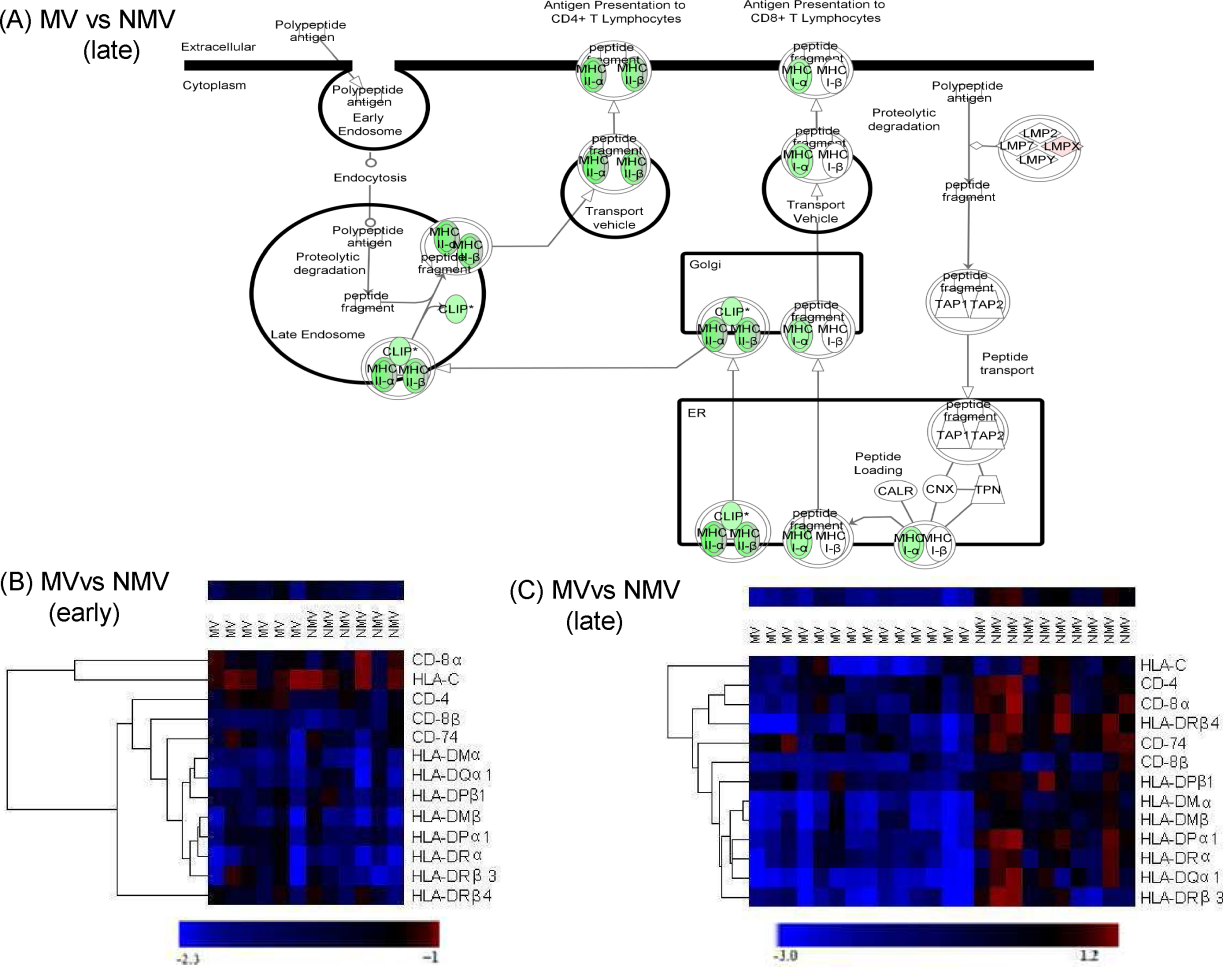
**Antigen presentation gene expression analysis**. **(a) **IPA canonical pathway modeling of the antigen presentation pathway in the late period (from day 9 in the course of the disease) using the microarray analysis of gene expression data from the peripheral blood mononuclear cells of the patients (red, upregulated; green, downregulated). **(b **and **c) **One-way hierarchical clustering of those genes selected by IPA (red, upregulated; blue, downregulated) in the early and late phases of the disease.

Comparison of gene expression profiles between MV and NMV patients in the early phase of the disease revealed the absence of differentially expressed genes between both groups. On the other hand, comparisons in the late phase of the disease revealed 4559 genes differentially expressed between MV and NMV patients (*P *< 0.05, FDR = 0.06). IPA analysis identified in this late phase a significant depression of a group of intracellular signaling pathways important for the development of the antiviral immune response in the most severe group of patients (MV) compared to NMV group (Figure 2). A number of genes involved in antigen presentation (Figure 3), B cell development (Figure S2 in Additional file 4), CD28 signaling in T helper cells (Figure S3 in Additional file 5), granzyme B signaling (Figure S4 in Additional file 6), T helper cell differentiation, protein ubiquitination, dendritic cell maturation, apoptosis and B-cell receptor (BCR) signaling were differentially regulated between these two groups and significantly lower in the MV group. On the contrary, the MV group showed significantly higher expression of the IL-6 and IL-10 signaling pathways (Figures S5 and S6 in Additional files 7 and 8). Tables S2-S9 (Additional files 9, 10, 11, 12, 13, 14, 15 and 16) show the genes differentially expressed between groups classified by individual pathway. IPA did not identify any signaling pathway differentially expressed between the seven MV patients with fatal outcome and the remaining five who survived the infection. Expression levels of HLA-DMA, HLA-DRB3, HLA-DRB4, HLA-DQA1, HLA-DRA1, HLA-DMB, HLA-DPA1, CD4, CD8A and CD8B showed a negative correlation with viral load in pharynx during the late phase of disease (*P *< 0.05, *r *≤ (-0.4)). These genes, with the exception of HLA-DRB4, correlated positively with O_2 _saturation in this phase (*P *< 0.05, *r *≥ 0.5). They showed also a negative association with the SOFA score for severity, as also did CD74 and HLA-C (*P *< 0.05, *r *≤ (-0.5)).

### Immune mediators profiling

**Figure 4:**
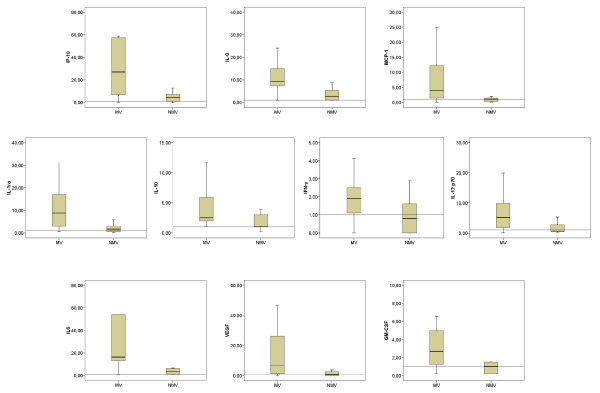
**Immune mediator levels in the late phase (from day 9 in the course of the disease)**. Boxplots show the ratios MV/[control median] and NMV/[control median]. All the comparisons showed significant differences at the level *P *< 0.05.

NMV and MV groups did not show major differences in the cytokine and chemokine profiles during the early phase of disease (Table S10 in Additional file 17). However, in the late phase, MV patients showed significantly higher levels of the chemokines IP-10 (CXCL10), IL8 (CXCL8) and MCP-1 (CCL2) (Figure 4 and Table S11 in Additional file 18). During the late phase, MV patients showed higher levels of two key Th1 cytokines (IL-12p70 and IFN-γ), IL6 (a Th17 related cytokine) and also two cellular growth factors (VEGF, GM-CSF) than the less severe patients (NMV). Also during the late phase, MV patients showed increased levels of two immunomodulatory cytokines (IL-10, IL-1ra) (Figure 4 and Table S11 in Additional file 18). Levels of IP-10, IL-6, and IL-8 showed a positive correlation with viral load in pharynx during the course of the disease (*P *< 0.05, *r *coefficient < 0.5). IL-6, IL-8, IL-10, IL-15, IL-12p70, GM-CSF and IFN-γ showed a positive correlation with the SOFA score for severity (*P *< 0.05, *r *coefficient < 0.5). IL-10 levels showed an inverse association with HLA-DRB3, HLADPB1 and CD74 expression levels; IL-1ra showed an inverse association with HLA-C expression levels (*P *< 0.05, *r *≤ (-0.5)). IFN-α, IFN-λ (IL-28) and IL-23 were undetectable in the vast majority of the patients in both groups along the course of the disease.

## Discussion

Here we examined host immune responses in severe patients requiring admission to the ICU. On the basis of the presence of anti-p2009A(H1N1) antibodies, we were able to identify two phases of disease in severe patients: an early phase characterized by the absence of antibodies (innate immunity phase) and a later phase defined by the presence of circulating anti-p2009A(H1N1) antibodies (adaptive immunity phase). Analysis of gene expression and cytokine profiles led to the characterization of signatures that are associated with disease severity and poor outcome in the late phase.

The impaired expression of a number of MHC class II (DM, DP, DQ, DR), MHC class I (HLA-C) genes, of T cell receptor-associated genes (CD4, CD8A, CD8B1) and also of a group of genes participating in dendritic cell maturation (CCR7, CD1C, IL18) points to the existence of a defective antigen presentation in the most severe group of patients (those who needed mechanical ventilation, MV) in the late phase of adaptive immunity. An adequate antigen presentation is needed to develop an effective adaptive immunity to influenza viruses [16]. Under some circumstances, changes affecting antigen presentation more strongly impact viral kinetics in the host than other viral or immune factors [16]. Disruption of antigen presentation prevents an effective adaptive immune response. Evidence on the potential role of an altered antigen presentation on the development of an appropriated adaptive response against the virus comes from the impaired expression of a group of genes pivotal to the activation and function of both T and B cells observed in the MV group in the late phase. Defective expression of CXCR5, MHC class II molecules, IL12RB1, IL21R and IL6R supports an impaired T helper cell differentiation signaling in this group of patients. Poor expression of CD4, FYN, GRB2, MHC class II molecules, ITPR3, MALT1, NFATC1, NFATC3, PDPK1, PIK3R1 and PLCG1 genes indicates a disruption in CD28 signaling in T helper cells, which is needed for effective primary T-cell expansion [17]. Impaired expression of DFFA, ENDOG, NUMA1, PARP1 and PRKDC affects granzyme B signaling. This pathway is involved in the induction of apoptosis in virus-infected cells by cytotoxic T lymphocytes (CTLs) [18]. Impaired T helper cell differentiation, CD28 and granzyme B signalling, along with the poor expression of T cell receptor associated genes (CD4, CD8A, CD8B1), supports a defective T cell response during the phase of adaptive immunity in the MV group. Moreover, the poor expression of genes related to B cell development and B cell receptor signaling (CD79A, CD79B, IL7R, MHC class II molecules, ABL1, CAMK2 D, MALT1, INPP5 D, HRAS, GRB2) points to an altered B cell function during this key period of the host response to the virus.

Additional clues on the existence of a defective adaptive response in severe pandemic influenza come from the impaired expression of a group of genes participating in the apoptosis signaling pathway (AIFM1, BIRC3, CAPN1, CAPN7, CAPNS1, CASP6, DFFA, ENDOG, HRAS, PARP1, PLCG1, TP 53). Since apoptosis is a recognized antiviral mechanism [19], a defect in apoptosis could translate into poor control of the virus. Additionally, defective expression of several ubiquitin-conjugating enzymes and ubiquitin-specific peptidases demonstrates that ubiquitination is also affected in severe pandemic influenza during the phase of adaptive response. Ubiquitination regulates the development of many phases of the immune response, including its initiation, propagation and termination [20]. The alteration of this pathway in severe pandemic influenza could affect in consequence all the steps needed for the development of an appropriate response to the virus. The role of steroids or immunosuppressor drugs in the genesis of the impaired adaptive response should be very limited, since none of the patients of the most severe group were under immunosuppressor treatment by the admission date. In addition, as detailed in Table 1, the proportion of patients under steroid treatment at the moment of sample collection during the hospitalization period was very similar in both groups of patients (41.6% for MV and 57.1% in NMV); in consequence, steroid treatment should affect similarly both groups in terms of modulation of the immune response. The ability showed by the vast majority of the patients in the MV group to produce specific antibodies indicates that antibody generation was insufficient to overcome the infection. Our results on gene expression support a defect in cellular immunity on the basis of the poor control of the virus in this group. It is well known that T helper and CTL responses play a determinant role in the containment of influenza once infection has occurred [21-23]. Our group is now designing further studies aimed at clarifying the participation of cellular responses in the severe disease caused by p2009A(H1N1).

On the other hand, patients of the MV group showed higher expression levels of those genes participating of the IL-6 and IL-10 canonical pathways during the phase of adaptive immunity. These pathways play opposite roles: proinflammatory and anti-inflammatory, respectively. In addition, serum levels of both IL-6 and IL-10 proteins are the highest in the MV group in this phase group which showed also elevated levels of chemokines, Th1 cytokines and growth factors. The presence of hypercytokinemia has been recently reported during infection by p2009A(H1N1) [8]. It has been described also during fatal H5N1 disease, severe SARS [6,7], acute RSV bronchiolitis [24] and sepsis [25]. Positive association between chemokines, cytokines and viral load in our study evidences that they are markers of ongoing viral replication as previously observed in SARS and H5N1 infection [6,12]. The high levels of the immunomodulatory molecules IL-1ra and IL-10 could represent an attempt to prevent cytokine-driven inflammatory damage or alternatively a virus-induced evasion mechanism [26-29]. The positive correlation observed between IL-10, viral load and SOFA, and the negative correlations between this cytokine and the expression levels of the genes participating in the antigen presentation pathway, supports the role of this mediator in favoring viral replication. As detailed in Table 1, bacterial superinfections took place not in the early but in the late course of the disease. This supports the role of the impaired adaptive response and the release of immunosuppressory cytokines in the increased incidence of bacterial superinfection observed in severe disease following infection by p2009A(H1N1) [2].

## Conclusions

**Figure 5:**
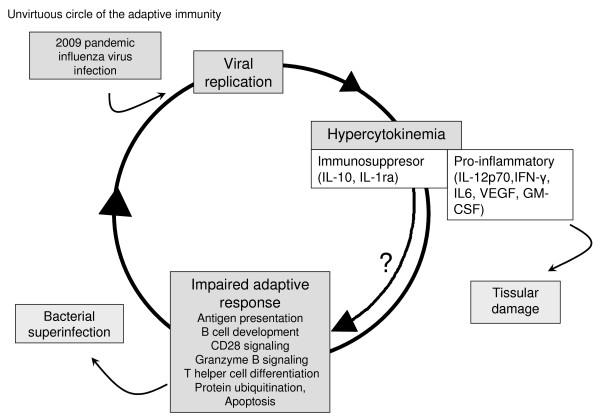
**Host adaptive immunity deficiency (HAID) model in severe pandemic influenza**. The picture shows the unvirtuous circle of the response to the virus.

Our findings suggest a state of host adaptive immunity deficiency (HAID) in the patients with severe pandemic influenza, leading to an unremitting cycle of viral replication and innate cytokine-chemokine release (Figure 5). This scenario of HAID resembles to the concept of immunoparalysis described for sepsis [30]. Interruption of this deleterious cycle may lead to improved disease outcome.

## Key messages

• The association between host immune responses and clinical outcome in severe pandemic 2009 influenza is poorly known. The potential for the use of gene signatures to better assess the immunopathology and clinical management of severe viral infections has been widely demonstrated in the past.

• Previous studies examining host gene expression profiles in other emerging viruses such as SARS-associated coronavirus, suggest severe disease is characterized by a malfunction of the switch from innate to adaptive immunity in response to the virus. Similar to severe infections caused by H5N1 influenza virus, dysregulated cytokine secretion has been described in severe cases of p2009A(H1N1).

• Pandemic H1N1 patients with severe respiratory disease and poor outcomes are characterized by an impaired activation of those genes participating in the development of the antiviral adaptive response and by persistence of the virus in the respiratory tract. These findings suggest a state of HAID that resembles the concept of immunoparalysis described for sepsis.

• HAID coexists with a persistent release of cytokines in those patients with the poorest outcomes.

• These results support the idea that HAID would lead to an unremitting cycle of viral replication and innate cytokine-chemokine release. Interruption of this deleterious cycle with antiviral and/or immunomodulatory therapies may lead to improved disease outcome.

## Abbreviations

FGF-b: human fibroblast growth factor-basic; G-CSF granulocyte colony-stimulating factor; GM-CSF: granulocyte macrophage colony-stimulating factor; HAID: host adaptive immunity deficiency; IFN-α: interferon-α; IFN-γ: interferon-γ; IL-1RA: interleukin 1 receptor antagonist; IP-10: interferon-inducible protein-10; MCP-1: monocyte chemoattractant protein-1; MIP-1α: macrophage inflammatory protein-1α; MIP-1β: macrophage inflammatory protein-1β; nvH1N1: new variant of H1N1 influenza virus; PDGF-BB: platelet-derived growth factor; RSV: respiratory syncytial virus; SARS: severe acute respiratory syndrome; SOFA: sepsis-related organ failure assessment; TNF-α: tumor necrosis factor α; VEGF: vascular endothelial growth factor.

## Competing interests

The authors declare that they have no competing interests.

## Authors' contributions

JFBM, IML, JR, ROL assisted in the design of the study, coordinated patient recruitment, analyzed and interpreted the data, and assisted in writing the paper. DK assisted in the design of the study, analyzed and interpreted the data, and assisted in writing the paper. AA, TP, MAM, MCG, VF, DV, BN, Sro, CC performed the virology works, RA, GLC, FMS and LR were in charge of the bioinformatic analysis. PR, LS, AL, DA, EM, MJGS, MG, SA, CL, PM, JB, FG, FB supervised clinical aspects, participated in patient recruitment and assisted in the analysis, interpretation of data, and writing the report. DB and DCN developed HAI assays and assisted in the analysis of data. LR, LX, and VI carried out microarray data, cytokine profiling and sample processing. SRE assisted in the statistical analysis.

## Supplementary Material

Additional file 1 **Methods additional material**. Additional information on viral load quantification, microarrays and number of samples analyzed is provided here. Click here for file

Additional file 2 **Figure S1: qPCR validation of microarray results**. A significant positive correlation was observed between the levels of expression obtained by qPCR and microarray analysis. Results are shown as adimensional units. Click here for file

Additional file 3 **Table S1: Clinical characteristics of fatal cases in MV group**. Click here for file

Additional file 4 **Figure S2: IPA modeling of the B cell development signaling pathway**. Expression in MV < NMV, represented in green. Click here for file

Additional file 5 **Figure S3: IPA modeling of the CD28 signaling pathway in T helper cells**. Expression in MV < NMV, represented in green. Click here for file

Additional file 6 **Figure S4: IPA modeling of the Granzyme B signaling pathway**. Expression in MV < NMV, represented in green. Click here for file

Additional file 7 **Figure S5: IPA modeling of the IL-6 signaling pathway**. Expression in MV > NMV, represented in red. Click here for file

Additional file 8 **Figure S6: IPA modeling of the IL-10 signaling pathway**. Expression in MV > NMV, represented in red. Click here for file

Additional file 9 **Table S2: Gene expression levels by intracellular signaling pathway (antigen presentation pathway, B cell development, granzyme B signaling)**. Difference between MV-NMV gene expression means is shown for each gene in the late period (from day 9 in the course of the disease). Click here for file

Additional file 10 **Table S3: Gene expression levels by intracellular signaling pathway (CD28 signaling in T helper cells)**. Difference between MV-NMV gene expression means is shown for each gene in the late period (from day 9 in the course of the disease). Click here for file

Additional file 11 **Table S4: Gene expression levels by intracellular signaling pathway (dendritic cell maturation)**. Difference between MV-NMV gene expression means is shown for each gene in the late period (from day 9 in the course of the disease). Click here for file

Additional file 12 **Table S5: Gene expression levels by intracellular signaling pathway (T helper cell differentiation)**. Difference between MV-NMV gene expression means is shown for each gene in the late period (from day 9 in the course of the disease). Click here for file

Additional file 13 **Table S6: Gene expression levels by intracellular signaling pathway (protein ubiquitination pathway)**. Difference between MV-NMV gene expression means is shown for each gene in the late period (from day 9 in the course of the disease). Click here for file

Additional file 14 **Table S7: Gene expression levels by intracellular signaling pathway (apoptosis signaling)**. Difference between MV-NMV gene expression means is shown for each gene in the late period (from day 9 in the course of the disease). Click here for file

Additional file 15 **Table S8: Gene expression levels by intracellular signaling pathway (B cell receptor signaling)**. Difference between MV-NMV gene expression means is shown for each gene in the late period (from day 9 in the course of the disease). Click here for file

Additional file 16 **Table S9: Gene expression levels by intracellular signaling pathway (IL-6, IL-10 signaling)**. Difference between MV-NMV gene expression means is shown for each gene in the late period (from day 9 in the course of the disease). Click here for file

Additional file 17 **Table S10: Comparison of immune mediator levels, early period (before day 9 in the course of the disease)**. Data are represented as median (interquartile range) of the ratios MV/(control median) and NMV/(control median). *Significant differences at the level *P *< 0.05. (n.s.), nonsignificant differences. IFN-α, IFN-λ (IL-28) and IL-23 were undetectable in the vast majority of the patients in both groups along the course of the disease. Click here for file

Additional file 18 **Table S11: Comparison of immune mediator levels, late period (from day 9 in the course of the disease)**. Data are represented as median (interquartile range) of the ratios MV/(control median) and NMV/(control median). *P < 0.05. n.s., nonsignificant differences. IFN-α, IFN-λ(IL-28) and IL-23 were undetectable in the vast majority of the patients in both groups along the course of the disease. Click here for file
