# Supplementary material for: Host adaptive immunity deficiency in severe pandemic influenza
Source: Crit Care. 2010 Sep 14;14(5):R167. doi: 10.1186/cc9259 (PMC3219262; doi:10.1186/cc9259)
Supplement: Additional file 1 — Methods additional material. Additional information on viral load quantification, microarrays and number of samples analyzed is provided here. [file cc9259-S1.doc]

**Methods Addtional Material**

**Number of samples analyzed:**

6 samples from 4 MV patients and 6 samples from 6 NMV patients were assayed for gene expression profiles by using microarrays in the early phase. In the late phase, 14 samples from 9 MV patients, and 10 samples from 6 NMV patients were proccessed for microarray analysis. 8 serum samples from 4 MV patients and 7 serum samples from 6 NMV patients were profiled for cytokine levels in the early phase, for 17 samples from 9 MV patients and 13 samples from 6 NMV patients in the late phase. In the early phase of the disease, viral load was meassured in 7 pharyngeal swabs from 4 MV patients and in 7 pharyngeal swabs from 6 NMV patients. In the late phase, viral load was meassured in 15 pharyngeal swabs from 9 MV patients and also in 11 pharyngeal swabs from 6 NMV.

**Viral load quantification method supp information**

A first single real time RT-PCR was performed for influenza A viral load quantification with a set of primers and probes specific for the influenza virus matrix protein (M1) gene ^1^. A standard graph was constructed from the threshold cycle values obtained from serially diluted cRNA control. cRNA control was manufactured from PCR product cloned into plasmid (pGEM-T Easy Vector System, Promega). This plasmid contained a T7 RNA polymerase promoter upstream of the insert to drive transcription by this enzyme. Previous to be in vitro transcribed using MEGAscript Kit (Ambion), the plasmid was linearized with a restriction enzyme (SpeI, New England Biolabs) downstream of the insert. With an additional DNase treatment any residual DNA was removed from RNA transcript. Finally RNA was puryfied using MEGAclear Kit (Ambion). The concentration and purity of the RNA control was calculated by measuring OD260/280. Known RNA control was serially diluted ten-fold in sterile water for use in real time PCR. The limit of detection was 1.86 log10 RNA copies/reaction with a 98.3% efficiency (data not shown). In order to modulate the viral load detected in relation with the quality of the samples collected, a second single real time PCR was performed to detect and quantitate genomic copies of the human RNase P gene. (WHO, 2009b) Since there are 2 copies of Rnase P gene per human cell, the amount of cells in the sample can be inferred using a serial dilution of human genomic DNA of known concentration obtained from human peripheral blood mononuclear cells as standard. The limit of detection of this assay was 9 cells with a 95.9% efficiency (data not shown).

1. Suwannakarn K, Payungporn S, Chieochansin T, et al. Typing (A/B) and subtyping (H1/H3/H5) of influenza A viruses by multiplex real-time RT-PCR assays. *J Virol Methods* 2008;**152**(1-2)**:**25-31.

**Microarray methods supp information**

RNA was stabilized and purified using PAXgene blood collection tubes and PAXgene Blood RNA kits (Qiagen, Mississauga, ON, Canada) following manufacturers instructions. RNA quality was assessed using a UV spectrophotometer and by formaldehyde gel electrophoresis. Samples containing an insufficient amount of RNA or containing RNA of bad quality were not assayed. Total RNA was amplified using Illumina TotalPrep RNA amplification Kit (Ambion, Austin, TX) per the manufacturer's instruction. cRNA (1.5 µg) was labelled and hybridized to Sentrix Human-6 v2 Expression BeadChip (Illumina, San Diego, CA) and scanned on Illumina BeadStation 500GX. Raw data collection was performed using Illumina GenomeStudio V2010.1 software. Data analysis were performed in the University Health Network (UHN), Toronto, Canada and also in the Medical Bioinformatics Lab-National Center of Microbiology, Majadahonda, Spain. The data sets were pre-processed with quantile normalization, variance stabilization, log2 transformation, and normalization against the data sets for four healthy controls excluding elements with median signal intensities of <6 log2 units across samples. Student’s t tests were used to identify genes significantly differentially expressed (P < 0.05 and >2-fold) between the two groups. Benjamini-Hochberg correction was employed to assess the occurrence of false positives by calculating the false discovery ratio (FDR).
