# Supplementary figures and images for: Host adaptive immunity deficiency in severe pandemic influenza
Source: Crit Care. 2010 Sep 14;14(5):R167. doi: 10.1186/cc9259 (PMC3219262; doi:10.1186/cc9259)

**HLA-DM $\alpha$**

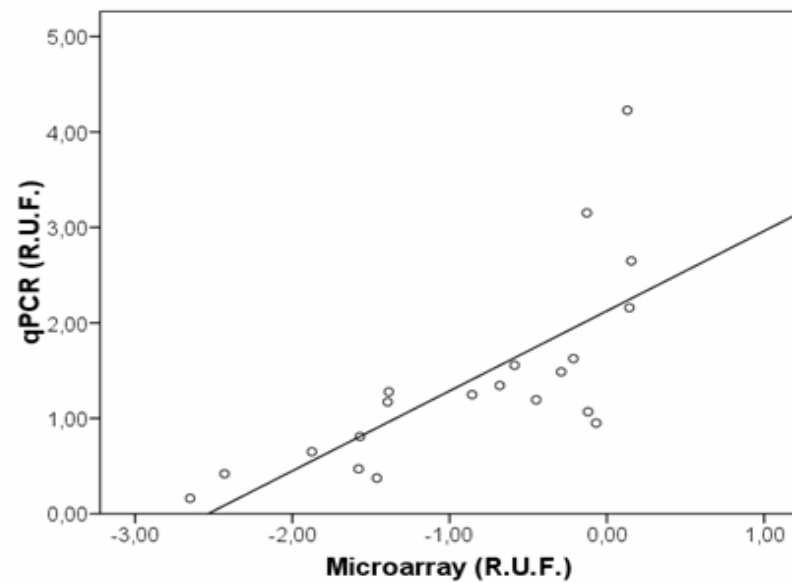

**HLA-DP $\alpha$ 1**

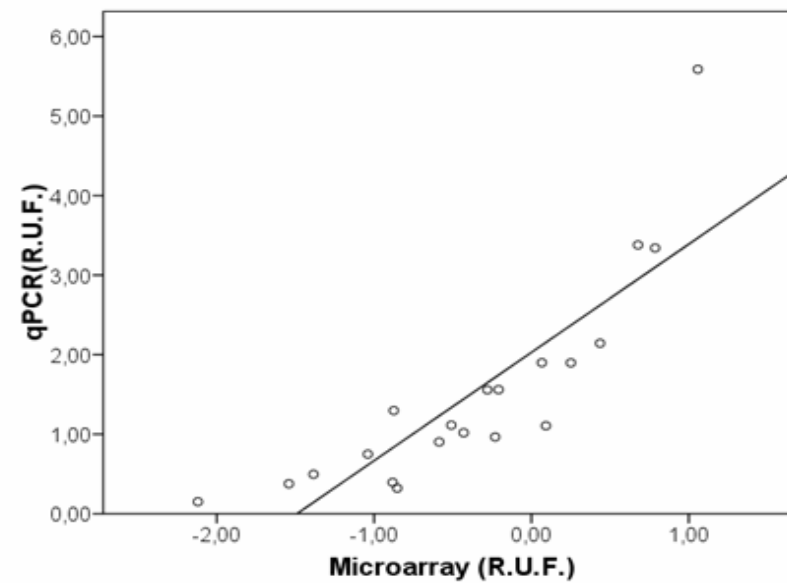

**CD8 $\alpha$**

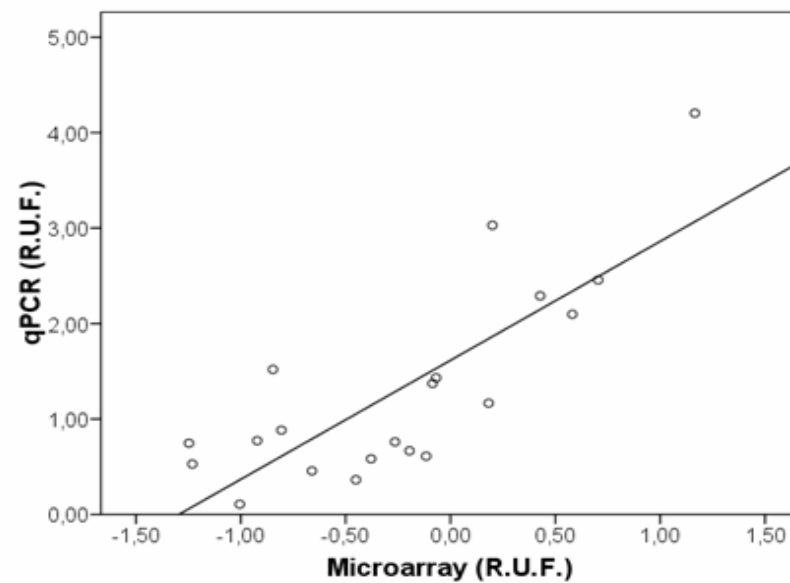

Supplement: Additional file 2 — Figure S1: qPCR validation of microarray results. A significant positive correlation was observed between the levels of expression obtained by qPCR and microarray analysis. Results are shown as adimensional units. [file cc9259-S2.pdf]

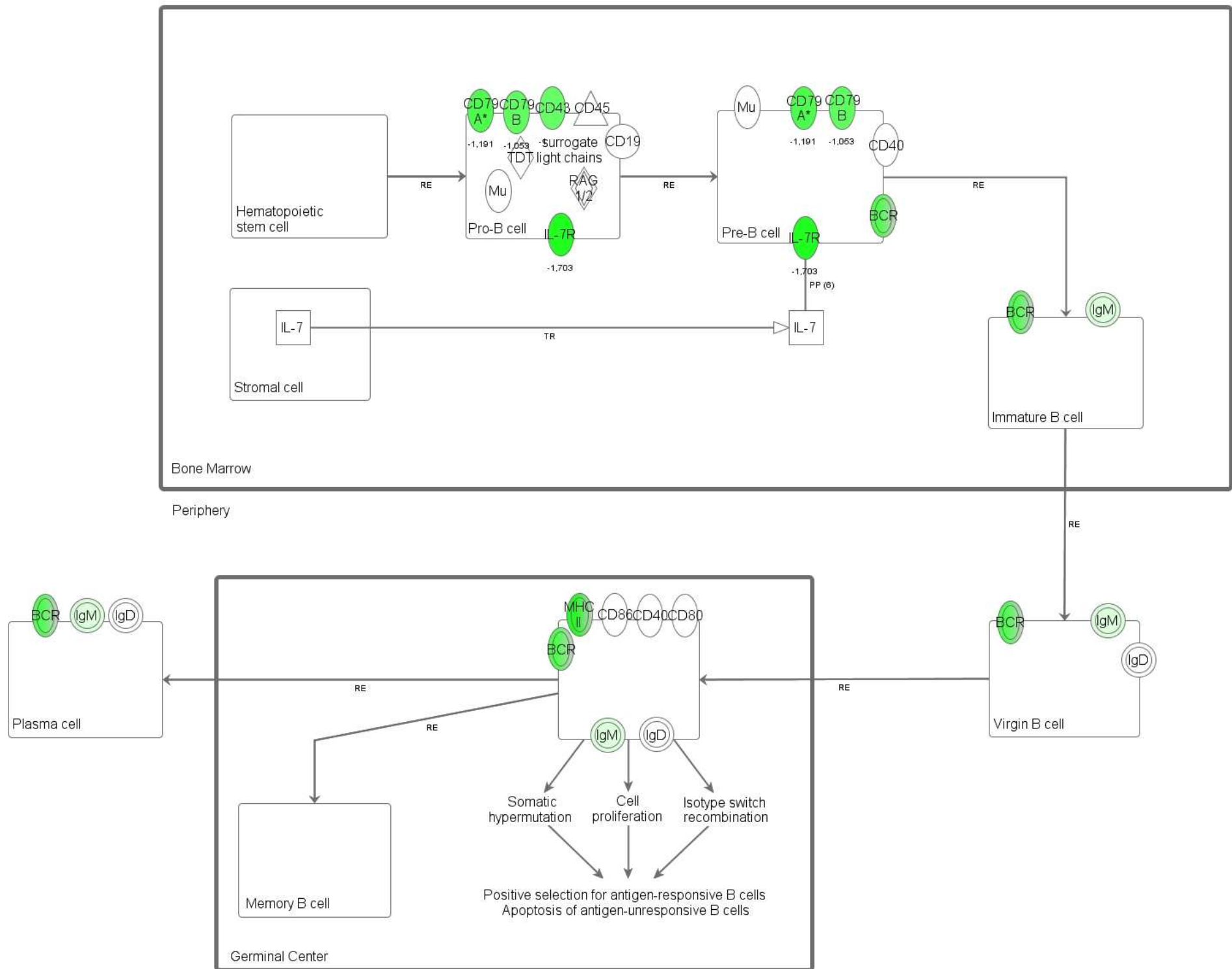

Supplement: Additional file 4 — Figure S2: IPA modeling of the B cell development signaling pathway. Expression in MV < NMV, represented in green. [file cc9259-S4.pdf]

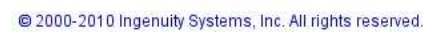

Supplement: Additional file 5 — Figure S3: IPA modeling of the CD28 signaling pathway in T helper cells. Expression in MV < NMV, represented in green. [file cc9259-S5.pdf]

# Granzyme B Signaling

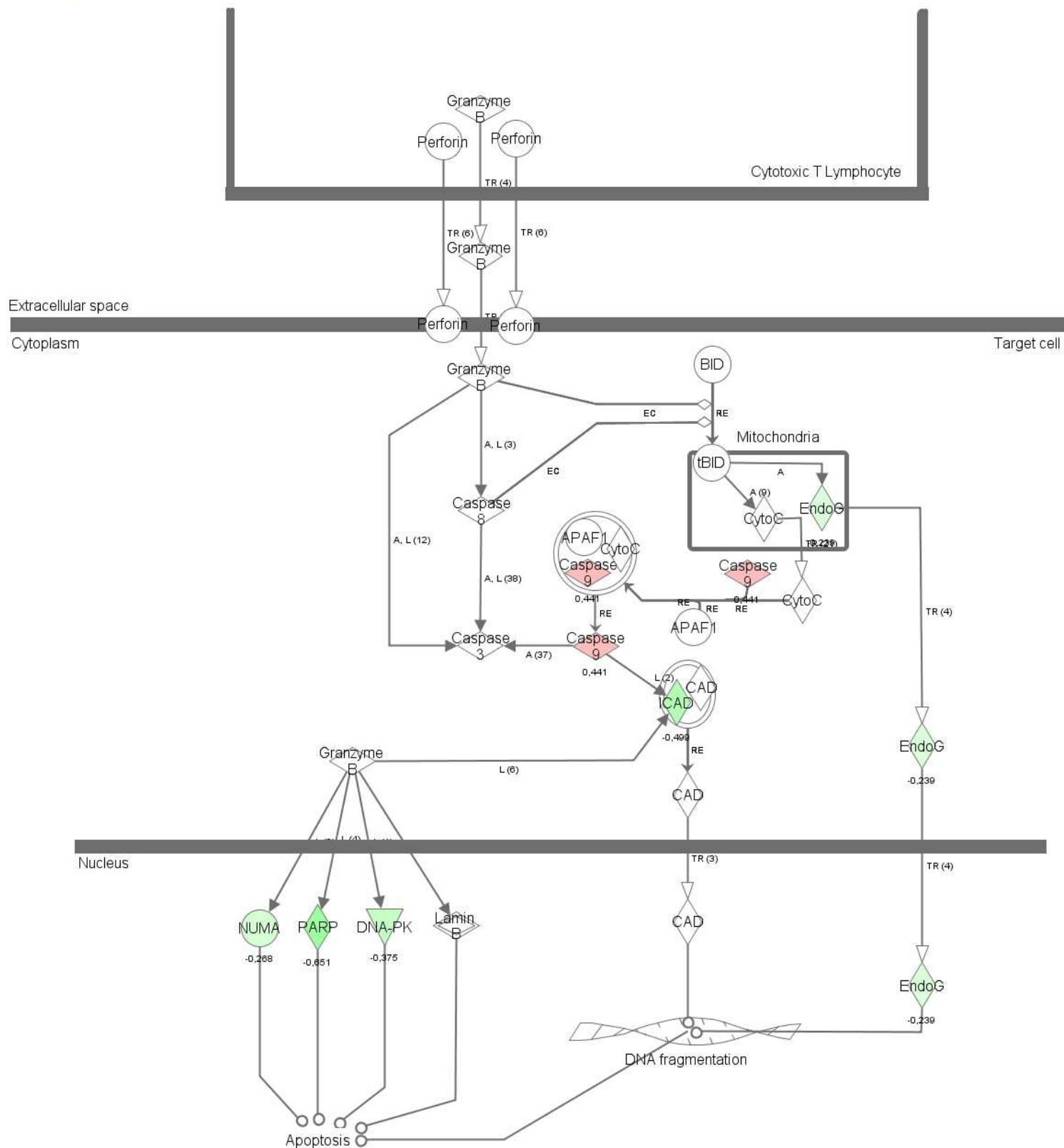

Supplement: Additional file 6 — Figure S4: IPA modeling of the Granzyme B signaling pathway. Expression in MV < NMV, represented in green. [file cc9259-S6.pdf]

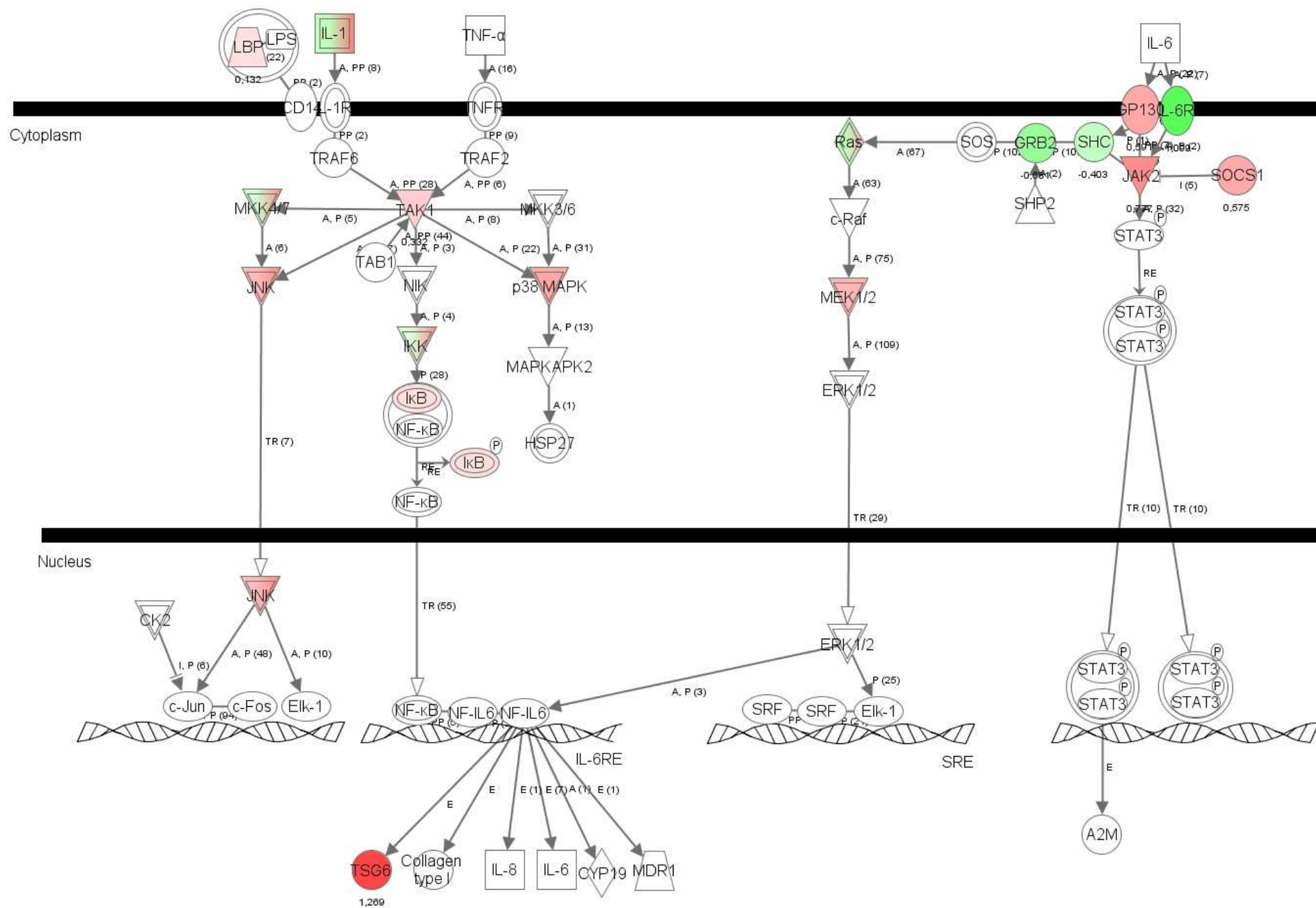

Supplement: Additional file 7 — Figure S5: IPA modeling of the IL-6 signaling pathway. Expression in MV > NMV, represented in red. [file cc9259-S7.pdf]

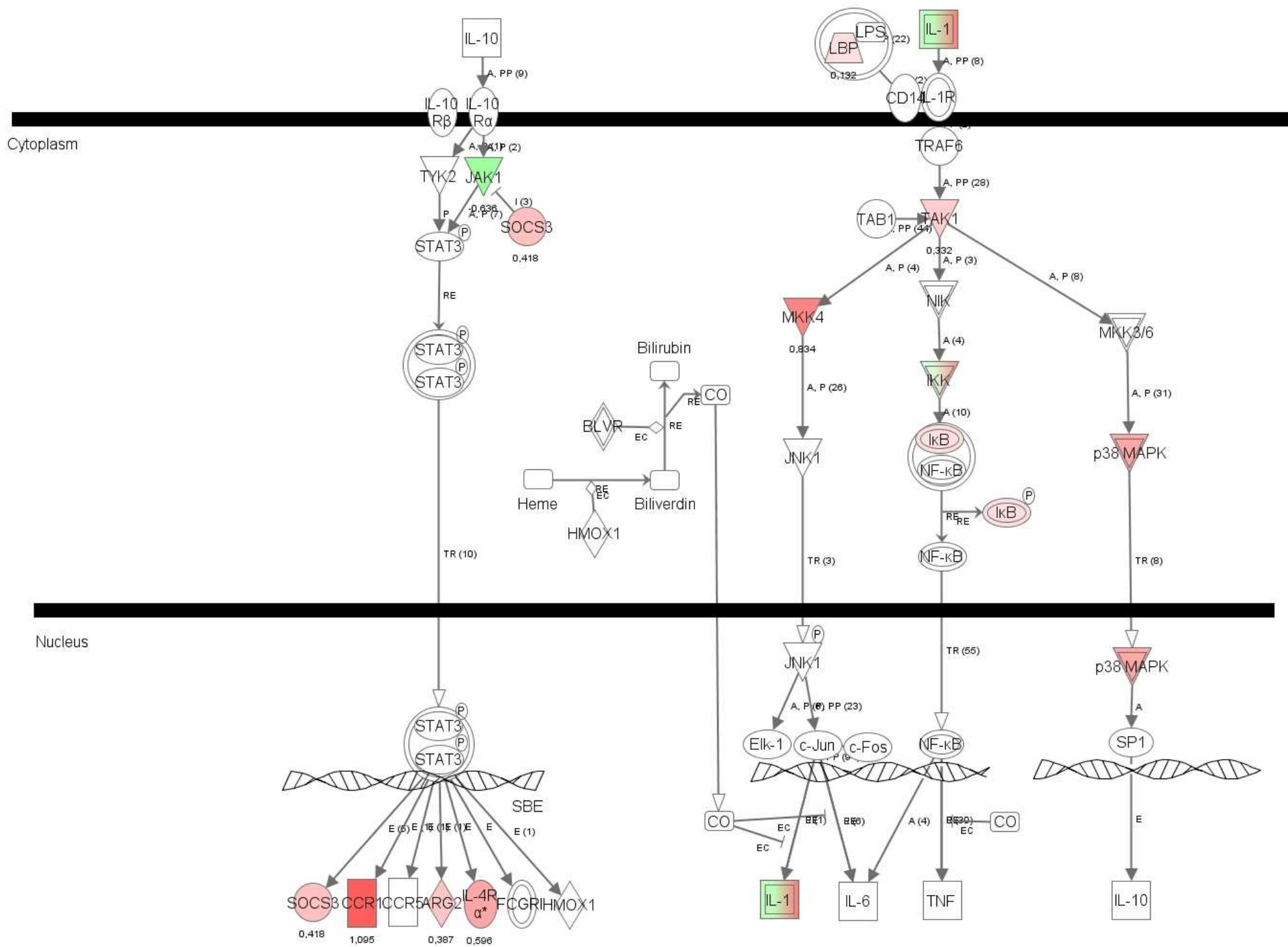

Supplement: Additional file 8 — Figure S6: IPA modeling of the IL-10 signaling pathway. Expression in MV > NMV, represented in red. [file cc9259-S8.pdf]
