# Supplementary material for: Host adaptive immunity deficiency in severe pandemic influenza
Source: Crit Care. 2010 Sep 14;14(5):R167. doi: 10.1186/cc9259 (PMC3219262; doi:10.1186/cc9259)
Supplement: Additional file 3 — Table S1: Clinical characteristics of fatal cases in MV group. [file cc9259-S3.doc]

|  | **Sex** | **Age** | **Clinical antecedents** | **Days since onset to death** | **Bacterial infection**  **(days since ICU admission to first bacterial isolation)** | **Primary cause of death** |
| --- | --- | --- | --- | --- | --- | --- |
| **Case 1** | M | 30 | None | 23 | No | Respiratory failure |
| **Case 2** | F | 49 | Obesity,  Asthma  Liver Cirrhosis | 17 | S. aureus Enterococcus faecalis,  A. fumigatus  (2 days) | Multiorganic failure |
| **Case 3** | F | 29 | Pregnancy | 24 | No | Respiratory failure |
| **Case 4** | M | 39 | COPD Smoker Asthma Esquizophrenia  Obesity | 13 | Serratia marcenses  (7 days) | Multiorganic failure |
| **Case 5** | M | 44 | Smoker  Psicotic flares | 7 | No | Respiratory failure |
| **Case 6** | M | 54 | Neurological disease | 18 | No | Respiratory failure |
| **Case 7** | M | 40 | Smoker | 15 | Pseudomonas aeruginosa  (5 days) | Respiratory failure |
