# Supplementary material for: Host adaptive immunity deficiency in severe pandemic influenza
Source: Crit Care. 2010 Sep 14;14(5):R167. doi: 10.1186/cc9259 (PMC3219262; doi:10.1186/cc9259)
Supplement: Additional file 9 — Table S2: Gene expression levels by intracellular signaling pathway (antigen presentation pathway, B cell development, granzyme B signaling). Difference between MV-NMV gene expression means is shown for each gene in the late period (from day 9 in the course of the disease). [file cc9259-S9.doc]

| **Canonical Pathways** | **Gene Symbol** | **Entrez Gene Name** | **Log Ratio** | **Top Functions &**  **Diseases:** |
| --- | --- | --- | --- | --- |
| **Antigen Presentation Pathway** | CD74 | CD74 molecule, major histocompatibility complex, class II invariant chain | -0.711 | **Immunological Disease; Cell Morphology; Cellular Assembly and Organization** |
|  | HLA-C | major histocompatibility complex, class I, C | -0.753 |  |
|  | HLA-DMA | major histocompatibility complex, class II, DM alpha | -1.321 |  |
|  | HLA-DMB | major histocompatibility complex, class II, DM beta | -1.138 |  |
|  | HLA-DPA1 | major histocompatibility complex, class II, DP alpha 1 | -1.271 |  |
|  | HLA-DPB1 | major histocompatibility complex, class II, DP beta 1 | -0.574 |  |
|  | HLA-DQA1 | major histocompatibility complex, class II, DQ alpha 1 | -1.643 |  |
|  | HLA-DRA | major histocompatibility complex, class II, DR alpha | -0.88 |  |
|  | HLA-DRB3 | major histocompatibility complex, class II, DR beta 3 | -1.169 |  |
|  | HLA-DRB4 | major histocompatibility complex, class II, DR beta 4 | -1.455 |  |
|  | PSMB5 | proteasome (prosome, macropain) subunit, beta type, 5 | 0.545 |  |
|  | CD4 | CD4 molecule | -0.574 |  |
|  | CD8A | CD8 molecule (alpha chain) | -0.834 |  |
|  | CD8B1 | CD8 molecule (beta 1 chain) | -0.494 |  |
| **B Cell Development** | CD79A | CD79a molecule, immunoglobulin-associated alpha | -1.191 | **Inflammatory Response; Cellular Development; Hematological System Development and Function** |
|  | CD79B | CD79b molecule, immunoglobulin-associated beta | -1.053 |  |
|  | HLA-DMA | major histocompatibility complex, class II, DM alpha | -1.321 |  |
|  | HLA-DMB | major histocompatibility complex, class II, DM beta | -1.138 |  |
|  | HLA-DQA1 | major histocompatibility complex, class II, DQ alpha 1 | -1.643 |  |
|  | HLA-DQB1 | major histocompatibility complex, class II, DQ beta 1 | -1.249 |  |
|  | HLA-DRA | major histocompatibility complex, class II, DR alpha | -0.88 |  |
|  | HS.548415 | immunoglobulin heavy constant gamma 1 (G1m marker) | -0.187 |  |
|  | IL7R | interleukin 7 receptor | -1.703 |  |
|  | SPN | sialophorin | -1.014 |  |
| **Granzyme B Signaling** | CASP9 | caspase 9, apoptosis-related cysteine peptidase | 0.441 | **Rapid induction of target cell apoptosis by CTL in cell-mediated immune response** |
|  | DFFA | DNA fragmentation factor, 45kDa, alpha polypeptide | -0.499 |  |
|  | ENDOG | endonuclease G | -0.239 |  |
|  | NUMA1 | nuclear mitotic apparatus protein 1 | -0.268 |  |
|  | PARP1 | poly (ADP-ribose) polymerase 1 | -0.651 |  |
|  | PRKDC | protein kinase, DNA-activated, catalytic polypeptide | -0.375 |  |
