# Supplementary material for: Host adaptive immunity deficiency in severe pandemic influenza
Source: Crit Care. 2010 Sep 14;14(5):R167. doi: 10.1186/cc9259 (PMC3219262; doi:10.1186/cc9259)
Supplement: Additional file 10 — Table S3: Gene expression levels by intracellular signaling pathway (CD28 signaling in T helper cells). Difference between MV-NMV gene expression means is shown for each gene in the late period (from day 9 in the course of the disease). [file cc9259-S10.doc]

| **Canonical Pathways** | **Gene Symbol** | **Entrez Gene Name** | **Log Ratio** | **Top Functions &**  **Diseases:** |
| --- | --- | --- | --- | --- |
| **CD28 Signaling in T Helper Cells** | ARPC3 | actin related protein 2/3 complex. subunit 3. 21kDa | 0.343 | **Cell-mediated Immune Response; Cellular Development; Cellular Function and Maintenance** |
|  | ARPC1A | actin related protein 2/3 complex. subunit 1A. 41kDa | 0.498 |  |
|  | CARD11 | caspase recruitment domain family. member 11 | -1.161 |  |
|  | CD4 | CD4 molecule | -0.574 |  |
|  | CDC42 | cell division cycle 42 (GTP binding protein. 25kDa) | 0.48 |  |
|  | CHUK | conserved helix-loop-helix ubiquitous kinase | 0.783 |  |
|  | FCER1G | Fc fragment of IgE. high affinity I. receptor for; gamma polypeptide | 1.123 |  |
|  | FYN | FYN oncogene related to SRC. FGR. YES | -1.064 |  |
|  | GRB2 | growth factor receptor-bound protein 2 | -0.681 |  |
|  | HLA-DMA | major histocompatibility complex. class II. DM alpha | -1.321 |  |
|  | HLA-DMB | major histocompatibility complex. class II. DM beta | -1.138 |  |
|  | HLA-DQA1 | major histocompatibility complex. class II. DQ alpha 1 | -1.643 |  |
|  | HLA-DQB1 | major histocompatibility complex. class II. DQ beta 1 | -1.249 |  |
|  | HLA-DRA | major histocompatibility complex. class II. DR alpha | -0.88 |  |
|  | IKBKB | inhibitor of kappa light polypeptide gene enhancer in B-cells. kinase beta | -0.236 |  |
|  | IL2 | interleukin 2 | 0.102 |  |
|  | ITPR3 | inositol 1.4.5-triphosphate receptor. type 3 | -0.893 |  |
|  | LCP2 | lymphocyte cytosolic protein 2 (SH2 domain containing leukocyte protein of 76kDa) | 0.609 |  |
|  | MALT1 | mucosa associated lymphoid tissue lymphoma translocation gene 1 | -0.533 |  |
|  | MAP2K1 | mitogen-activated protein kinase kinase 1 | 0.494 |  |
|  | MAP2K4 | mitogen-activated protein kinase kinase 4 | 0.834 |  |
|  | MAPK9 | mitogen-activated protein kinase 9 | 0.432 |  |
|  | NFATC1 | nuclear factor of activated T-cells. cytoplasmic. calcineurin-dependent 1 | -0.425 |  |
|  | NFATC3 | nuclear factor of activated T-cells. cytoplasmic. calcineurin-dependent 3 | -0.435 |  |
|  | NFKBIB | nuclear factor of kappa light polypeptide gene enhancer in B-cells inhibitor. beta | 0.208 |  |
|  | PDPK1 | 3-phosphoinositide dependent protein kinase-1 | -0.608 |  |
|  | PIK3R1 | phosphoinositide-3-kinase. regulatory subunit 1 (alpha) | -0.673 |  |
|  | PLCG1 | phospholipase C. gamma 1 | -0.736 |  |
|  | PPP3CA | protein phosphatase 3. catalytic subunit. alpha isozyme | 0.366 |  |
|  | PPP3CB | protein phosphatase 3. catalytic subunit. beta isozyme | 0.629 |  |
|  | HS.546375 | T cell receptor delta locus | -1.87 |  |
|  | WAS | Wiskott-Aldrich syndrome (eczema-thrombocytopenia) | -0.282 |  |
