# Supplementary material for: Host adaptive immunity deficiency in severe pandemic influenza
Source: Crit Care. 2010 Sep 14;14(5):R167. doi: 10.1186/cc9259 (PMC3219262; doi:10.1186/cc9259)
Supplement: Additional file 11 — Table S4: Gene expression levels by intracellular signaling pathway (dendritic cell maturation). Difference between MV-NMV gene expression means is shown for each gene in the late period (from day 9 in the course of the disease). [file cc9259-S11.doc]

| **Canonical Pathways** | **Gene Symbol** | **Entrez Gene Name** | **Log Ratio** | **Top Functions &**  **Diseases:** |
| --- | --- | --- | --- | --- |
| **Dendritic cell maduration** | ATF2 | activating transcription factor 2 | 0.379 | **T cell activation.** **induction of immune responses** |
|  | CCR7 | chemokine (C-C motif) receptor 7 | -0.856 |  |
|  | CD58 | CD58 molecule | 0.982 |  |
|  | CD1C | CD1c molecule | -0.188 |  |
|  | CHUK | conserved helix-loop-helix ubiquitous kinase | 0.783 |  |
|  | HS.516646 | cAMP responsive element binding protein 1 | -0.761 |  |
|  | CREB5 | cAMP responsive element binding protein 5 | 0.857 |  |
|  | FCER1G | Fc fragment of IgE. high affinity I. receptor for; gamma polypeptide | 1.123 |  |
|  | FCGR1A | Fc fragment of IgG. high affinity Ia. receptor (CD64) | 1.14 |  |
|  | FSCN1 | fascin homolog 1. actin-bundling protein (Strongylocentrotus purpuratus) | -0.583 |  |
|  | HLA-C | major histocompatibility complex. class I. C | -0.753 |  |
|  | HLA-DMA | major histocompatibility complex. class II. DM alpha | -1.321 |  |
|  | HLA-DMB | major histocompatibility complex. class II. DM beta | -1.138 |  |
|  | HLA-DQA1 | major histocompatibility complex. class II. DQ alpha 1 | -1.643 |  |
|  | HLA-DQB1 | major histocompatibility complex. class II. DQ beta 1 | -1.249 |  |
|  | HLA-DRA | major histocompatibility complex. class II. DR alpha | -0.88 |  |
|  | HLA-DRB3 | major histocompatibility complex. class II. DR beta 3 | -1.169 |  |
|  | HLA-DRB4 | major histocompatibility complex. class II. DR beta 4 | -1.455 |  |
|  | ICAM1 | intercellular adhesion molecule 1 | 0.307 |  |
|  | IFNAR1 | interferon (alpha. beta and omega) receptor 1 | 0.743 |  |
|  | HS.548415 | immunoglobulin heavy constant gamma 1 (G1m marker) | -0.187 |  |
|  | IKBKB | inhibitor of kappa light polypeptide gene enhancer in B-cells. kinase beta | -0.236 |  |
|  | IL18 | interleukin 18 (interferon-gamma-inducing factor) | -0.43 |  |
|  | IL1F6 | interleukin 1 family. member 6 (epsilon) | 0.122 |  |
|  | IL1RN | interleukin 1 receptor antagonist | 0.91 |  |
|  | HS.572649 | interleukin 23. alpha subunit p19 | -1.067 |  |
|  | JAK2 | Janus kinase 2 | 0.777 |  |
|  | MAP2K4 | mitogen-activated protein kinase kinase 4 | 0.834 |  |
|  | MAPK9 | mitogen-activated protein kinase 9 | 0.432 |  |
|  | MAPK14 | mitogen-activated protein kinase 14 | 0.593 |  |
|  | MYD88 | myeloid differentiation primary response gene (88) | 0.774 |  |
|  | NFKBIB | nuclear factor of kappa light polypeptide gene enhancer in B-cells inhibitor. beta | 0.208 |  |
|  | PIK3R1 | phosphoinositide-3-kinase. regulatory subunit 1 (alpha) | -0.673 |  |
|  | TLR4 | toll-like receptor 4 | 0.595 |  |
|  | HS.546375 | T cell receptor delta locus | -1.87 |  |
|  | TYROBP | TYRO protein tyrosine kinase binding protein | 0.316 |  |
