# Supplementary material for: Host adaptive immunity deficiency in severe pandemic influenza
Source: Crit Care. 2010 Sep 14;14(5):R167. doi: 10.1186/cc9259 (PMC3219262; doi:10.1186/cc9259)
Supplement: Additional file 12 — Table S5: Gene expression levels by intracellular signaling pathway (T helper cell differentiation). Difference between MV-NMV gene expression means is shown for each gene in the late period (from day 9 in the course of the disease). [file cc9259-S12.doc]

| **Canonical Pathways** | **Gene Symbol** | **Entrez Gene Name** | **Log Ratio** | **Top Functions &**  **Diseases:** |
| --- | --- | --- | --- | --- |
| **T helper cell differentiation** | BCL6 | B-cell CLL/lymphoma 6 | 1.038 | **Immunological Disease; Cell-mediated Immune Response;** **Cytokine production** |
|  | CXCR5 | chemokine (C-X-C motif) receptor 5 | -0.765 |  |
|  | FCER1G | Fc fragment of IgE. high affinity I. receptor for; gamma polypeptide | 1.123 |  |
|  | HLA-DMA | major histocompatibility complex. class II. DM alpha | -1.321 |  |
|  | HLA-DMB | major histocompatibility complex. class II. DM beta | -1.138 |  |
|  | HLA-DQA1 | major histocompatibility complex. class II. DQ alpha 1 | -1.643 |  |
|  | HLA-DQB1 | major histocompatibility complex. class II. DQ beta 1 | -1.249 |  |
|  | HLA-DRA | major histocompatibility complex. class II. DR alpha | -0.88 |  |
|  | IFNGR1 | interferon gamma receptor 1 | 0.832 |  |
|  | IL2 | interleukin 2 | 0.102 |  |
|  | IL4 | interleukin 4 | 0.074 |  |
|  | IL18 | interleukin 18 (interferon-gamma-inducing factor) | -0.43 |  |
|  | IL12RB1 | interleukin 12 receptor. beta 1 | -0.662 |  |
|  | IL18R1 | interleukin 18 receptor 1 | 1.809 |  |
|  | IL21R | interleukin 21 receptor | -0.5 |  |
|  | IL4R | interleukin 4 receptor | 0.596 |  |
|  | IL6R | interleukin 6 receptor | -1.099 |  |
|  | HS.570988 | interleukin 6 signal transducer (gp130. oncostatin M receptor) | 0.591 |  |
|  | RORC | RAR-related orphan receptor C | -0.309 |  |
|  | TGFBR1 | transforming growth factor. beta receptor 1 | 0.293 |  |
|  | TGFBR2 | transforming growth factor. beta receptor II (70/80kDa) | 0.584 |  |
|  | HS.546375 | T cell receptor delta locus | -1.87 |  |
| **Protein Ubiquitination Pathway** | AMFR | autocrine motility factor receptor | 0.772 | **Cell-mediated Immune Response; Cellular Development; Cellular Function and Maintenance** |
|  | ANAPC1 | anaphase promoting complex subunit 1 | -0.525 |  |
|  | ANAPC2 | anaphase promoting complex subunit 2 | -0.641 |  |
|  | ANAPC11 | anaphase promoting complex subunit 11 | -0.135 |  |
|  | BIRC3 | baculoviral IAP repeat-containing 3 | -0.36 |  |
|  | 38961 | Cas-Br-M (murine) ecotropic retroviral transforming sequence | -1.186 |  |
|  | FBXW7 | F-box and WD repeat domain containing 7 | -0.631 |  |
|  | HLA-C | major histocompatibility complex. class I. C | -0.753 |  |
|  | PAN2 | PAN2 poly(A) specific ribonuclease subunit homolog (S. cerevisiae) | -0.232 |  |
|  | PSMA1 | proteasome (prosome. macropain) subunit. alpha type. 1 | -0.508 |  |
|  | PSMA2 | proteasome (prosome. macropain) subunit. alpha type. 2 | 0.399 |  |
|  | PSMB5 | proteasome (prosome. macropain) subunit. beta type. 5 | 0.545 |  |
|  | PSMB10 | proteasome (prosome. macropain) subunit. beta type. 10 | -0.483 |  |
|  | PSMC3 | proteasome (prosome. macropain) 26S subunit. ATPase. 3 | -0.428 |  |
|  | PSMC4 | proteasome (prosome. macropain) 26S subunit. ATPase. 4 | -0.62 |  |
