# Supplementary material for: Host adaptive immunity deficiency in severe pandemic influenza
Source: Crit Care. 2010 Sep 14;14(5):R167. doi: 10.1186/cc9259 (PMC3219262; doi:10.1186/cc9259)
Supplement: Additional file 13 — Table S6: Gene expression levels by intracellular signaling pathway (protein ubiquitination pathway). Difference between MV-NMV gene expression means is shown for each gene in the late period (from day 9 in the course of the disease). [file cc9259-S13.doc]

| **Canonical Pathways** | **Gene Symbol** | **Entrez Gene Name** | **Log Ratio** | **Top Functions &**  **Diseases:** |
| --- | --- | --- | --- | --- |
| **Protein Ubiquitination Pathway** | AMFR | autocrine motility factor receptor | 0.772 | **Cell-mediated Immune Response; Cellular Development; Cellular Function and Maintenance** |
|  | ANAPC1 | anaphase promoting complex subunit 1 | -0.525 |  |
|  | ANAPC2 | anaphase promoting complex subunit 2 | -0.641 |  |
|  | ANAPC11 | anaphase promoting complex subunit 11 | -0.135 |  |
|  | BIRC3 | baculoviral IAP repeat-containing 3 | -0.36 |  |
|  | 38961 | Cas-Br-M (murine) ecotropic retroviral transforming sequence | -1.186 |  |
|  | FBXW7 | F-box and WD repeat domain containing 7 | -0.631 |  |
|  | HLA-C | major histocompatibility complex. class I. C | -0.753 |  |
|  | PAN2 | PAN2 poly(A) specific ribonuclease subunit homolog (S. cerevisiae) | -0.232 |  |
|  | PSMA1 | proteasome (prosome. macropain) subunit. alpha type. 1 | -0.508 |  |
|  | PSMA2 | proteasome (prosome. macropain) subunit. alpha type. 2 | 0.399 |  |
|  | PSMB5 | proteasome (prosome. macropain) subunit. beta type. 5 | 0.545 |  |
|  | PSMB10 | proteasome (prosome. macropain) subunit. beta type. 10 | -0.483 |  |
|  | PSMC3 | proteasome (prosome. macropain) 26S subunit. ATPase. 3 | -0.428 |  |
|  | PSMC4 | proteasome (prosome. macropain) 26S subunit. ATPase. 4 | -0.62 |  |
|  | PSMC5 | proteasome (prosome. macropain) 26S subunit. ATPase. 5 | -0.656 |  |
|  | PSMD1 | proteasome (prosome. macropain) 26S subunit. non-ATPase. 1 | 0.364 |  |
|  | PSMD6 | proteasome (prosome. macropain) 26S subunit. non-ATPase. 6 | 0.458 |  |
|  | PSMD7 | proteasome (prosome. macropain) 26S subunit. non-ATPase. 7 | 0.238 |  |
|  | PSMD8 | proteasome (prosome. macropain) 26S subunit. non-ATPase. 8 | -0.315 |  |
|  | PSMD10 | proteasome (prosome. macropain) 26S subunit. non-ATPase. 10 | 0.489 |  |
|  | PSMD12 | proteasome (prosome. macropain) 26S subunit. non-ATPase. 12 | -0.347 |  |
|  | SKP1A | S-phase kinase-associated protein 1 | 0.556 |  |
|  | STUB1 | STIP1 homology and U-box containing protein 1 | -0.449 |  |
|  | TCEB1 | transcription elongation factor B (SIII). polypeptide 1 (15kDa. elongin C) | 0.336 |  |
|  | THOP1 | thimet oligopeptidase 1 | -0.396 |  |
|  | UBE2D2 | ubiquitin-conjugating enzyme E2D 2 (UBC4/5 homolog. yeast) | -0.442 |  |
|  | UBE2D4 | ubiquitin-conjugating enzyme E2D 4 (putative) | -0.312 |  |
|  | UBE2F | ubiquitin-conjugating enzyme E2F (putative) | 0.874 |  |
|  | UBE2G2 | ubiquitin-conjugating enzyme E2G 2 (UBC7 homolog. yeast) | -0.735 |  |
|  | UBE2L3 | ubiquitin-conjugating enzyme E2L 3 | -0.122 |  |
|  | UBE2L6 | ubiquitin-conjugating enzyme E2L 6 | -0.086 |  |
|  | UBE2V1 | ubiquitin-conjugating enzyme E2 variant 1 | 0.169 |  |
|  | USP3 | ubiquitin specific peptidase 3 | 0.368 |  |
|  | USP4 | ubiquitin specific peptidase 4 (proto-oncogene) | -0.427 |  |
|  | USP11 | ubiquitin specific peptidase 11 | -0.212 |  |
|  | USP14 | ubiquitin specific peptidase 14 (tRNA-guanine transglycosylase) | -0.727 |  |
|  | USP15 | ubiquitin specific peptidase 15 | 0.725 |  |
|  | USP18 | ubiquitin specific peptidase 18 | 0.188 |  |
|  | USP22 | ubiquitin specific peptidase 22 | -0.475 |  |
|  | USP32 | ubiquitin specific peptidase 32 | 0.866 |  |
|  | USP33 | ubiquitin specific peptidase 33 | -0.451 |  |
