# Supplementary material for: Host adaptive immunity deficiency in severe pandemic influenza
Source: Crit Care. 2010 Sep 14;14(5):R167. doi: 10.1186/cc9259 (PMC3219262; doi:10.1186/cc9259)
Supplement: Additional file 14 — Table S7: Gene expression levels by intracellular signaling pathway (apoptosis signaling). Difference between MV-NMV gene expression means is shown for each gene in the late period (from day 9 in the course of the disease). [file cc9259-S14.doc]

| **Canonical Pathways** | **Gene Symbol** | **Entrez Gene Name** | **Log Ratio** | **Top Functions &**  **Diseases:** |
| --- | --- | --- | --- | --- |
| **Apoptosis signaling** | ACIN1 | apoptotic chromatin condensation inducer 1 | 0.461 | **Cell death;** **Cell termination; Homeostasis ; Development; Lymphocyte interactions** |
|  | AIFM1 | apoptosis-inducing factor. mitochondrion-associated. 1 | -0.239 |  |
|  | BIRC3 | baculoviral IAP repeat-containing 3 | -0.36 |  |
|  | CAPN1 | calpain 1. (mu/I) large subunit | -0.447 |  |
|  | CAPN7 | calpain 7 | -0.353 |  |
|  | CAPNS1 | calpain. small subunit 1 | -0.525 |  |
|  | CASP2 | caspase 2. apoptosis-related cysteine peptidase | 0.344 |  |
|  | CASP6 | caspase 6. apoptosis-related cysteine peptidase | -0.77 |  |
|  | CASP9 | caspase 9. apoptosis-related cysteine peptidase | 0.441 |  |
|  | CHUK | conserved helix-loop-helix ubiquitous kinase | 0.783 |  |
|  | DFFA | DNA fragmentation factor. 45kDa. alpha polypeptide | -0.499 |  |
|  | ENDOG | endonuclease G | -0.239 |  |
|  | FAS | Fas (TNF receptor superfamily. member 6) | 0.376 |  |
|  | HRAS | v-Ha-ras Harvey rat sarcoma viral oncogene homolog | -0.442 |  |
|  | HTRA2 | HtrA serine peptidase 2 | 0.367 |  |
|  | IKBKB | inhibitor of kappa light polypeptide gene enhancer in B-cells. kinase beta | -0.236 |  |
|  | KRAS | v-Ki-ras2 Kirsten rat sarcoma viral oncogene homolog | 0.5 |  |
|  | MAP2K1 | mitogen-activated protein kinase kinase 1 | 0.494 |  |
|  | MAP2K4 | mitogen-activated protein kinase kinase 4 | 0.834 |  |
|  | MAP2K7 | mitogen-activated protein kinase kinase 7 | -0.314 |  |
|  | NFKBIB | nuclear factor of kappa light polypeptide gene enhancer in B-cells inhibitor. beta | 0.208 |  |
|  | PARP1 | poly (ADP-ribose) polymerase 1 | -0.651 |  |
|  | PLCG1 | phospholipase C. gamma 1 | -0.736 |  |
|  | TP53 | tumor protein p53 | -0.3 |  |
|  | XIAP | X-linked inhibitor of apoptosis | -0.332 |  |
