# Supplementary material for: Host adaptive immunity deficiency in severe pandemic influenza
Source: Crit Care. 2010 Sep 14;14(5):R167. doi: 10.1186/cc9259 (PMC3219262; doi:10.1186/cc9259)
Supplement: Additional file 15 — Table S8: Gene expression levels by intracellular signaling pathway (B cell receptor signaling). Difference between MV-NMV gene expression means is shown for each gene in the late period (from day 9 in the course of the disease). [file cc9259-S15.doc]

| **Canonical Pathways** | **Gene Symbol** | **Entrez Gene Name** | **Log Ratio** | **Top Functions &**  **Diseases:** |
| --- | --- | --- | --- | --- |
| **B cell receptor signaling** | ABL1 | c-abl oncogene 1. receptor tyrosine kinase | -0.342 | **Differentiation. survival. apoptosis. proliferation and tolerance** |
|  | ATF2 | activating transcription factor 2 | 0.379 |  |
|  | BCL6 | B-cell CLL/lymphoma 6 | 1.038 |  |
|  | CAMK2D | calcium/calmodulin-dependent protein kinase II delta | -0.379 |  |
|  | CD79A | CD79a molecule. immunoglobulin-associated alpha | -1.191 |  |
|  | CD79B | CD79b molecule. immunoglobulin-associated beta | -1.053 |  |
|  | CDC42 | cell division cycle 42 (GTP binding protein. 25kDa) | 0.48 |  |
|  | CHUK | conserved helix-loop-helix ubiquitous kinase | 0.783 |  |
|  | HS.516646 | cAMP responsive element binding protein 1 | -0.761 |  |
|  | CREB5 | cAMP responsive element binding protein 5 | 0.857 |  |
|  | GRB2 | growth factor receptor-bound protein 2 | -0.681 |  |
|  | HRAS | v-Ha-ras Harvey rat sarcoma viral oncogene homolog | -0.442 |  |
|  | IKBKB | inhibitor of kappa light polypeptide gene enhancer in B-cells. kinase beta | -0.236 |  |
|  | INPP5D | inositol polyphosphate-5-phosphatase. 145kDa | -0.4 |  |
|  | KRAS | v-Ki-ras2 Kirsten rat sarcoma viral oncogene homolog | 0.5 |  |
|  | MALT1 | mucosa associated lymphoid tissue lymphoma translocation gene 1 | -0.533 |  |
|  | MAP2K1 | mitogen-activated protein kinase kinase 1 | 0.494 |  |
|  | MAP2K4 | mitogen-activated protein kinase kinase 4 | 0.834 |  |
|  | MAP2K7 | mitogen-activated protein kinase kinase 7 | -0.314 |  |
|  | MAP3K4 | mitogen-activated protein kinase kinase kinase 4 | -0.414 |  |
|  | MAP3K7 | mitogen-activated protein kinase kinase kinase 7 | 0.332 |  |
|  | MAPK9 | mitogen-activated protein kinase 9 | 0.432 |  |
|  | MAPK14 | mitogen-activated protein kinase 14 | 0.593 |  |
|  | NFATC1 | nuclear factor of activated T-cells. cytoplasmic. calcineurin-dependent 1 | -0.425 |  |
|  | NFATC3 | nuclear factor of activated T-cells. cytoplasmic. calcineurin-dependent 3 | -0.435 |  |
|  | NFKBIB | nuclear factor of kappa light polypeptide gene enhancer in B-cells inhibitor. beta | 0.208 |  |
|  | PIK3R1 | phosphoinositide-3-kinase. regulatory subunit 1 (alpha) | -0.673 |  |
|  | POU2F2 | POU class 2 homeobox 2 | -1.617 |  |
|  | PPP3CA | protein phosphatase 3. catalytic subunit. alpha isozyme | 0.366 |  |
|  | PPP3CB | protein phosphatase 3. catalytic subunit. beta isozyme | 0.629 |  |
|  | PRKCB1 | protein kinase C. beta | 0.362 |  |
|  | PTEN | phosphatase and tensin homolog | 0.741 |  |
|  | RPS6KB2 | ribosomal protein S6 kinase. 70kDa. polypeptide 2 | -0.738 |  |
|  | SHC1 | SHC (Src homology 2 domain containing) transforming protein 1 | -0.403 |  |
|  | VAV2 | vav 2 guanine nucleotide exchange factor | -0.319 |  |
|  | VAV3 | vav 3 guanine nucleotide exchange factor | 0.676 |  |
