# Supplementary material for: Host adaptive immunity deficiency in severe pandemic influenza
Source: Crit Care. 2010 Sep 14;14(5):R167. doi: 10.1186/cc9259 (PMC3219262; doi:10.1186/cc9259)
Supplement: Additional file 16 — Table S9: Gene expression levels by intracellular signaling pathway (IL-6, IL-10 signaling). Difference between MV-NMV gene expression means is shown for each gene in the late period (from day 9 in the course of the disease). [file cc9259-S16.doc]

| **Canonical Pathways** | **Gene Symbol** | **Entrez Gene Name** | **Log Ratio** | **Top Functions &**  **Diseases:** |
| --- | --- | --- | --- | --- |
| **IL-6**  **Signaling** | CHUK | conserved helix-loop-helix ubiquitous kinase | 0.783 | **Gene Expression; Cellular Development; Cellular Growth and Proliferation** |
|  | GRB2 | growth factor receptor-bound protein 2 | -0.681 |  |
|  | HRAS | v-Ha-ras Harvey rat sarcoma viral oncogene homolog | -0.442 |  |
|  | IKBKB | inhibitor of kappa light polypeptide gene enhancer in B-cells. kinase beta | -0.236 |  |
|  | IL18 | interleukin 18 (interferon-gamma-inducing factor) | -0.43 |  |
|  | IL1F6 | interleukin 1 family. member 6 (epsilon) | 0.122 |  |
|  | IL1RN | interleukin 1 receptor antagonist | 0.91 |  |
|  | IL6R | interleukin 6 receptor | -1.099 |  |
|  | HS.570988 | interleukin 6 signal transducer (gp130. oncostatin M receptor) | 0.591 |  |
|  | JAK2 | Janus kinase 2 | 0.777 |  |
|  | KRAS | v-Ki-ras2 Kirsten rat sarcoma viral oncogene homolog | 0.5 |  |
|  | LBP | lipopolysaccharide binding protein | 0.132 |  |
|  | MAP2K1 | mitogen-activated protein kinase kinase 1 | 0.494 |  |
|  | MAP2K4 | mitogen-activated protein kinase kinase 4 | 0.834 |  |
|  | MAP2K7 | mitogen-activated protein kinase kinase 7 | -0.314 |  |
|  | MAP3K7 | mitogen-activated protein kinase kinase kinase 7 | 0.332 |  |
|  | MAPK9 | mitogen-activated protein kinase 9 | 0.432 |  |
|  | MAPK14 | mitogen-activated protein kinase 14 | 0.593 |  |
|  | NFKBIB | nuclear factor of kappa light polypeptide gene enhancer in B-cells inhibitor. beta | 0.208 |  |
|  | SHC1 | SHC (Src homology 2 domain containing) transforming protein 1 | -0.403 |  |
|  | SOCS1 | suppressor of cytokine signaling 1 | 0.575 |  |
|  | TNFAIP6 | tumor necrosis factor. alpha-induced protein 6 | 1.269 |  |
| **IL-10 Signaling** | ARG2 | arginase. type II | 0.387 | **Anti-inflammatory; Immunosuppersor; T cell tolerance** |
|  | CCR1 | chemokine (C-C motif) receptor 1 | 1.095 |  |
|  | CHUK | conserved helix-loop-helix ubiquitous kinase | 0.783 |  |
|  | IKBKB | inhibitor of kappa light polypeptide gene enhancer in B-cells. kinase beta | -0.236 |  |
|  | IL18 | interleukin 18 (interferon-gamma-inducing factor) | -0.43 |  |
|  | IL1F6 | interleukin 1 family. member 6 (epsilon) | 0.122 |  |
|  | IL1RN | interleukin 1 receptor antagonist | 0.91 |  |
|  | IL4R | interleukin 4 receptor | 0.596 |  |
|  | JAK1 | Janus kinase 1 | -0.636 |  |
|  | LBP | lipopolysaccharide binding protein | 0.132 |  |
|  | MAP2K4 | mitogen-activated protein kinase kinase 4 | 0.834 |  |
|  | MAP3K7 | mitogen-activated protein kinase kinase kinase 7 | 0.332 |  |
|  | MAPK14 | mitogen-activated protein kinase 14 | 0.593 |  |
|  | NFKBIB | nuclear factor of kappa light polypeptide gene enhancer in B-cells inhibitor. beta | 0.208 |  |
|  | SOCS3 | suppressor of cytokine signaling 3 | 0.418 |  |
