# Supplementary material for: Host adaptive immunity deficiency in severe pandemic influenza
Source: Crit Care. 2010 Sep 14;14(5):R167. doi: 10.1186/cc9259 (PMC3219262; doi:10.1186/cc9259)
Supplement: Additional file 17 — Table S10: Comparison of immune mediator levels, early period (before day 9 in the course of the disease). Data are represented as median (interquartile range) of the ratios MV/(control median) and NMV/(control median). *Significant differences at the level P < 0.05. (n.s.), nonsignificant differences. IFN-α, IFN-λ (IL-28) and IL-23 were undetectable in the vast majority of the patients in both groups along the course of the disease. [file cc9259-S17.doc]

|  | **MV/Control** | **NMV/Control** | **MV vs NMV**  **( *p* )** |
| --- | --- | --- | --- |
| **IL-1ra** | 3,26 [2,15] | 2,30 [26,69] | n.s. |
| **IL-9** | 14,00 [24,65] | 3,70 [9,20] | n.s. |
| **IL-15** | 1,00 [0,00] | 1,00 [0,00] | n.s. |
| **Eotaxin** | 0,51 [0,55] | 0,10 [0,30] | 0,042 |
| **FGFB** | 1,00 [21,05] | 1,00 [0,00] | n.s. |
| **IP-10** | 5,95 [82,50] | 61,80 [175,00] | n.s. |
| **MIP1a** | 1,00 [1,65] | 1,00 [0,00] | n.s. |
| **PDGFbb** | 0,65 [0,85] | 0,20 [0,72] | 0,031 |
| **RANTES** | 1,00 [0,00] | 1,00 [0,90] | n.s. |
| **VEGF** | 1,80 [3,47] | 0,68 [1,00] | 0,015 |
| **IL-1ß** | 1,00 [0,00] | 1,00 [0,00] | n.s. |
| **IL-6** | 8,40 [24,67] | 3,90 [755,30] | n.s. |
| **IL-8** | 5,15 [6,33] | 4,68 [17,80] | n.s. |
| **IL-7** | 1,80 [1,82] | 1,30 [0,70] | n.s. |
| **IL-17** | 2,70 [15,40] | 1,00 [0,00] | n.s. |
| **GCSF** | 4,32 [6,62] | 1,00 [7609,40] | n.s. |
| **MCP1** | 2,55 [21,27] | 1,20 [11,70] | n.s. |
| **MIP1ß** | 1,10 [0,82] | 0,70 [1,80] | n.s. |
| **IL-2** | 1,05 [0,10] | 1,10 [0,10] | n.s. |
| **IL-4** | 1,43 [1,29] | 0,40 [1,00] | n.s. |
| **IL-5** | 1,00 [0,00] | 1,00 [0,00] | n.s. |
| **IL-10** | 1,00 [2,60] | 1,40 [6,30] | n.s. |
| **IL-12p70** | 1,75 [2,95] | 0,60 [1,10] | n.s. |
| **GM-CSF** | 5,40 [19,64] | 1,00 [0,80] | 0,051 |
| **IFNγ** | 1,40 [0,77] | 1,00 [1,30] | n.s. |
| **TNFa** | 1,00 [0,88] | 1,00 [1,90] | n.s. |
| **IL-13** | 1,00 [0,90] | 1,00 [0,00] | n.s. |
| **IL-29** | 0,02 [0,97] | 0,02 [10,58] | n.s. |
| **Adiponectin** | 0,26 [1,06] | 1,00 [3,19] | n.s. |
| **Leptin** | 1,52 [4,72] | 0,83 [1,15] | n.s. |
| **TGFß** | 74,34 [222,48] | 57,51 [110,00] | n.s. |
