# Supplementary material for: Host adaptive immunity deficiency in severe pandemic influenza
Source: Crit Care. 2010 Sep 14;14(5):R167. doi: 10.1186/cc9259 (PMC3219262; doi:10.1186/cc9259)
Supplement: Additional file 18 — Table S11: Comparison of immune mediator levels, late period (from day 9 in the course of the disease). Data are represented as median (interquartile range) of the ratios MV/(control median) and NMV/(control median). *P < 0.05. n.s., nonsignificant differences. IFN-α, IFN-λ(IL-28) and IL-23 were undetectable in the vast majority of the patients in both groups along the course of the disease. [file cc9259-S18.doc]

|  | **MV/Control** | **NMV/Control** | **MV vs NMV**  **( *p* )** |
| --- | --- | --- | --- |
| **IL-1ra** | 8,80 [16,29] | 1,70 [2,65] | 0,003* |
| **IL-9** | 2,78 [2,60] | 2,00 [26,35] | n.s. |
| **IL-15** | 1,00 [1,30] | 1,00 [0,00] | n.s. |
| **Eotaxin** | 0,60 [0,72] | 0,30 [0,80] | 0,041* |
| **FGFB** | 1,00 [12,35] | 1,00 [1,45] | n.s. |
| **IP-10** | 26,90 [52,04] | 4,50 [9,30] | 0,011* |
| **MIP1a** | 1,00 [0,00] | 1,00 [0,00] | n.s. |
| **PDGFbb** | 1,00 [0,78] | 0,20 [0,85] | n.s. |
| **RANTES** | 1,00 [0,00] | 1,00 [0,90] | n.s. |
| **VEGF** | 7,00 [27,64] | 0,70 [3,15] | 0,020* |
| **IL-1ß** | 1,00 [0,00] | 1,00 [0,00] | n.s. |
| **IL-6** | 16,40 [79,95] | 3,60 [5,40] | 0,000* |
| **IL-8** | 9,30 [8,43] | 2,70 [4,45] | 0,000* |
| **IL-7** | 1,00 [3,85] | 1,00 [1,25] | n.s. |
| **IL-17** | 1,30 [4,65] | 1,00 [5,45] | n.s. |
| **GCSF** | 2,90 [3,20] | 1,10 [7,90] | n.s. |
| **MCP1** | 3,90 [11,56] | 1,10 [1,35] | 0,007* |
| **MIP1ß** | 1,30 [0,90] | 1,30 [1,40] | n.s. |
| **IL-2** | 1,00 [0,05] | 1,10 [0,10] | n.s. |
| **IL-4** | 1,40 [1,21] | 1,00 [1,45] | n.s. |
| **IL-5** | 1,00 [0,00] | 1,00 [0,00] | n.s. |
| **IL-10** | 2,50 [4,50] | 1,00 [2,20] | 0,015* |
| **IL-12p70** | 5,20 [10,55] | 0,60 [2,40] | 0,048* |
| **GM-CSF** | 2,80 [4,30] | 1,00 [2,40] | n.s. |
| **IFNγ** | 1,90 [1,50] | 0,80 [1,85] | 0,034* |
| **TNFa** | 1,00 [0,00] | 1,00 [0,55] | 0,044* |
| **IL-13** | 2,00 [2,30] | 2,30 [10,10] | n.s. |
| **IL-29** | 0,86 [0,49] | 0,02 [0,00] | 0,000* |
| **Adiponectin** | 0,77 [2,00] | 0,85 [3,24] | n.s. |
| **Leptin** | 0,77 [3,25] | 0,79 [0,97] | n.s. |
| **TGFß** | 9,02 [45,25] | 41,43 [98,30] | n.s. |
